# From mechanisms to anti-fibrotic drugs in hepatic stellate cell research: a global bibliometric analysis with patent and clinical perspectives (2000–2025)

**DOI:** 10.3389/fphar.2025.1734449

**Published:** 2025-12-10

**Authors:** Xiaowen Mao, Peng Chen, Palidan Wubur, Yi Liu, Yuan Zhao, Siwei Zhang, Na Guan, Bin Li

**Affiliations:** 1 Affiliated Urumqi Municipal Hospital of Traditional Chinese Medicine, Xinjiang Medical University, Urumqi, Xinjiang, China; 2 School of Basic Medical Science, Xi’an Medical University, Xi’an, Shaanxi, China; 3 Baoji Hospital Affiliated to Xi’an Medical University, Baoji, Shaanxi, China

**Keywords:** anti-fibrotic therapy, bibliometrics, clinical trials, hepatic stellate cells, liver fibrosis, patents, targeted delivery

## Abstract

**Background:**

Liver fibrosis (LF) is a progressive condition that can advance to cirrhosis and liver failure, posing a major global health burden. Hepatic stellate cells (HSCs) are central to LF pathogenesis via extracellular matrix (ECM) production and inflammatory regulation, and have been widely explored as therapeutic targets.

**Methods:**

We searched the Web of Science Core Collection (WoSCC), Scopus, and PubMed for English-language publications using the keywords “liver fibrosis” and “stellate cells.” Additionally, ClinicalTrials.gov was queried for clinical trials, and the Innojoy search engine was used for patents. Analyses were performed using CiteSpace (version 6.2.R4), VOSviewer, R, and Microsoft Excel to examine publication trends, collaboration and citation structures, keyword co-occurrence, clustering, citation bursts, and International Patent Classification (IPC) profiles.

**Results:**

From 2000 to 2025, annual publications increased from 3 to 50 (≈16.7-fold), totaling 1,042 papers; China led output (n = 672), followed by the United States (n = 162), spanning hepatology and pharmacology. Thirteen thematic clusters were identified across etiology, molecular mechanisms, and therapeutics/delivery, with targeted delivery and intervention emerging as the leading frontier. Burst terms highlighted sustained reliance on rodent *in vivo* models (rats/mice; carbon tetrachloride injury) alongside hepatocellular carcinoma-related signals. The patent landscape was dominated by therapeutic-use and small-molecule classes (A61P 1/16; A61K 31/), with expansion to specialized dosage forms and combination regimens (A61K 9/00; A61K 45/06) and multimodal platforms involving nucleic acids (C12N 15/113) and antibodies (C07K 16/18). Clinical trials shifted from early small molecule monotherapies to more diversified, combinable regimens.

**Conclusion:**

Integrating bibliometrics with patent and clinical landscapes, this study delineates an evolution from mechanism discovery to precision intervention in HSC - focused LF research. Future priorities include improving target/tissue specificity and advancing multimodal, patient stratified strategies to enhance translational efficiency.

## 1 Introduction

Liver fibrosis (LF) is a maladaptive wound-healing response to chronic or recurrent liver injury (Xu et al., 2023). If unchecked, it can progress to cirrhosis and liver failure and is among the leading causes of global morbidity and mortality, with an estimated ∼2 million liver-related deaths annually (Marcellin and Kutala, 2018; GBD, 2021 Diseases and Injuries Collaborators, 2024; Xiang et al., 2023). Major etiologies include alcohol-associated liver disease (ALD) (Babuta et al., 2024), nonalcoholic steatohepatitis (NASH) and obesity-related metabolic dysfunction (Thomas et al., 2024; Hernandez-Gea and Friedman, 2011), viral hepatitis (hepatitis B/C) (Fernandes et al., 2022), and parasitic infections (e.g., schistosomiasis) (Liu et al., 2023). Pathologically, LF is characterized by the transdifferentiation of quiescent HSCs into myofibroblasts, resulting in excessive extracellular matrix (ECM) deposition and abnormal connective tissue accumulation (Tacke and Trautwein, 2015). Early-stage LF reversal is closely linked to modulating aberrant HSCs activation (Kisseleva and Brenner, 2007). In the perisinusoidal space of Disse, quiescent HSCs are activated by profibrogenic factors such as platelet-derived growth factor (PDGF) (Wu et al., 2017; Gong et al., 2017), transforming growth factor-β (TGF-β) (Gharbia et al., 2022), and interleukin-17 (IL-17) (Kartasheva-Ebertz et al., 2022). This leads to loss of retinoid and lipid storage capacity (Niu et al., 2023; Jiang et al., 2015), enhanced proliferation, contractility, ECM upregulation, and modulation of matrix-degrading enzymes (Balmer and Blomhoff, 2002).

Following activation, HSCs undergo profound functional disruption, with the interstitial space filled by α-smooth muscle actin (α-SMA), collagens (types I, III, and VI), and other fibrotic markers (Udomsinprasert et al., 2020; Luangmonkong et al., 2023; Liang et al., 2023). Progressive LF results in scar formation, altered hepatic architecture, elasticity, and hemodynamics (Lee and Friedman, 2014; Liu et al., 2022), culminating in complications such as liver failure, portal hypertension-induced bleeding, ascites, and hepatocellular carcinoma (HCC) (Tsochatzis et al., 2014), often progressing to multi-organ dysfunction involving the liver, lungs, and kidneys (Urban et al., 2015).

Although numerous drugs have been explored in clinical trials, there are no Food and Drug Administration (FDA)-approved anti-fibrotic drugs specifically for treating liver fibrosis (Zhang et al., 2023). Pirfenidone and Nintedanib are the two most classic anti-fibrotic drugs, initially used for Idiopathic Pulmonary Fibrosis and later extended to other types of pulmonary fibrosis (Tanneberger et al., 2025; Kim et al., 2025). The current systemic treatment for anti-fibrotic drugs mainly relies on a multi-level integrated strategy, including etiology control (Marc et al., 2013), symptomatic support (such as anti-inflammatory and antioxidant) (Sanyal et al., 2010), targeted drugs (Zhao et al., 2024), and the combination of lifestyle interventions (Vilar-Gomez et al., 2015). Currently, liver transplantation remains the definitive treatment for advanced cirrhosis, yet outcomes are compromised by donor shortages, variable graft quality, and patient comorbidities, yielding suboptimal clinical efficacy (Haugen et al., 2019; Peng et al., 2024).

Despite these challenges, recent advances in hepatic microstructure and molecular biology have identified promising interventions. Activated HSCs (aHSCs) serve as key effector cells in LF and have emerged as a central focus in anti-fibrotic research, with recent studies in the field uncovering numerous promising interventions (El Taghdouini et al., 2015; Bu et al., 2018; Rockey, 2016). Advancements in nanocarrier technologies can significantly address the tissue-specific deficiencies of conventional drugs: PDGF-β peptide-modified chitosan nanoparticles enable targeted delivery of anti-TGF-β1-siRNA, reducing hepatic TGF-β1 levels by approximately 65% while overcoming ECM barriers (Mostafa et al., 2025); dual-ligand lipid carriers inhibit aHSC proliferation and reduce ECM deposition (Su et al., 2025); Bt/cRGDfK-R6-PPMsTK achieves membrane penetration and sequential, targeted release of payloads for specific aHSC delivery (Liu et al., 2025). In the reversal of LF involving HSCs, signaling pathway modulation represents a core mechanism: recent investigations have shown that retinoic acid-based nanoparticles delivering bortezomib suppress TGF-β1/Smad3 and nuclear factor-κB (NF-κB) gene expression in aHSCs (Siapoush et al., 2025); recombinant TMEM219-loaded flavinosomes target TGF-β1 downregulation via the TGFβ/IGFBP-3 signaling pathway (Mok et al., 2025); and formulations such as Dantao Fang and salvianolic acid B nanoparticles simultaneously ameliorate LF and portal hypertension by regulating pathways including cAMP/PKA/ROCK (Zeng et al., 2026; Wang et al., 2025). Subsequent multi-mechanistic synergistic strategies show further promise, with sorafenib nanovesicles combined with photothermal therapy reversing myofibroblast phenotypes and aHSC amplification (Xiang X. et al., 2025), while 3D LF models and red blood cell membrane encapsulation techniques provide robust support for clinical translation (Xiang L. et al., 2025; Huang et al., 2025).

Although recent studies in this field have proliferated, there remains a paucity of systematic reviews that integrate microscopic mechanisms with macroscopic developmental trends (Yao et al., 2020; Zou and Sun, 2021). Bibliometrics, through the construction of knowledge maps, co-citation/co-word analyses, and visualization techniques, enables the quantitative identification of research hotspots and collaboration networks (Huang et al., 2023).We integrate bibliometrics, patent landscaping, and clinical-trial mapping to chart HSC-targeted anti-fibrotic research across three dimensions—basic mechanisms, drug-development technologies, and clinical applications. Specifically, we (i) quantify global outputs, collaboration, and citation structures; (ii) resolve thematic clusters and their temporal evolution; (iii) align International Patent Classification (IPC) categories with therapeutic modalities; and (iv) profile the clinical pipeline by targets, phases, and endpoints—to identify disciplinary structures, technological hotspots, and translational bottlenecks and to inform research priorities and clinical translation pathways.

## 2 Materials and methods

### 2.1 Data sources

This bibliometric analysis used three primary data sources to comprehensively assess research trends, patents, and clinical trials on HSCs in LF: the Web of Science Core Collection (WoSCC), Scopus and PubMed for peer-reviewed publications; ClinicalTrials.gov (maintained by the U.S. National Library of Medicine at the National Institutes of Health) for registered clinical studies; and the Innojoy Patent Search Engine for global patent records. All data were retrieved on 15 June 2025, to ensure consistency across sources.

### 2.2 Search strategies

Searches were conducted without language restrictions, focusing on the period from 1 January 2000, to 15 June 2025 (or 31 December 2024, for clinical trials to align with completed records).

#### 2.2.1 Search formula: advanced search was performed using the following criteria

WoSCC search:TI = (“hepatic fibrosis” OR “liver fibrosis”) AND TI = (“hepatic stellate cells”); Scopus search: (TITLE (“hepatic fibrosis” OR “liver fibrosis”) AND TITLE (“hepatic stellate cells”)); PubMed search ((“hepatic fibrosis” [Title] OR “liver fibrosis” [Title]) AND (“hepatic stellate cells” [Title]))

Document types: Articles and Reviews;

Publication period: 1 January 2000, to 15 June 2025;

Data extracted: Publication volume, countries/regions, institutions, authors, citations, and keywords.

Data were downloaded in “plain text” format for analysis.

#### 2.2.2 ClinicalTrials.gov search: expert search mode was employed with

Conditions: (liver fibrosis OR hepatic fibrosis OR cirrhosis OR liver scarring) AND (“hepatic stellate cells” OR HSCs OR myofibroblast OR “activated HSCs”);

Study start date range: 1 January 2000, to 31 December 2024.

#### 2.2.3 Innojoy patent search

Patents with a Development Priority Index (DPI) ≥65 were targeted using:

Search formula: ((ABST = liver fibrosis AND ABST = hepatic stellate cells) and (IDX >= 90 or (IDX >= 80 and IDX < 90) or (IDX >= 70 and IDX < 80) or (IDX >= 65 and IDX < 70));

This ensured selection of high-relevance patents based on indexing scores.

### 2.3 Inclusion and exclusion criteria

**FIGURE 1:**
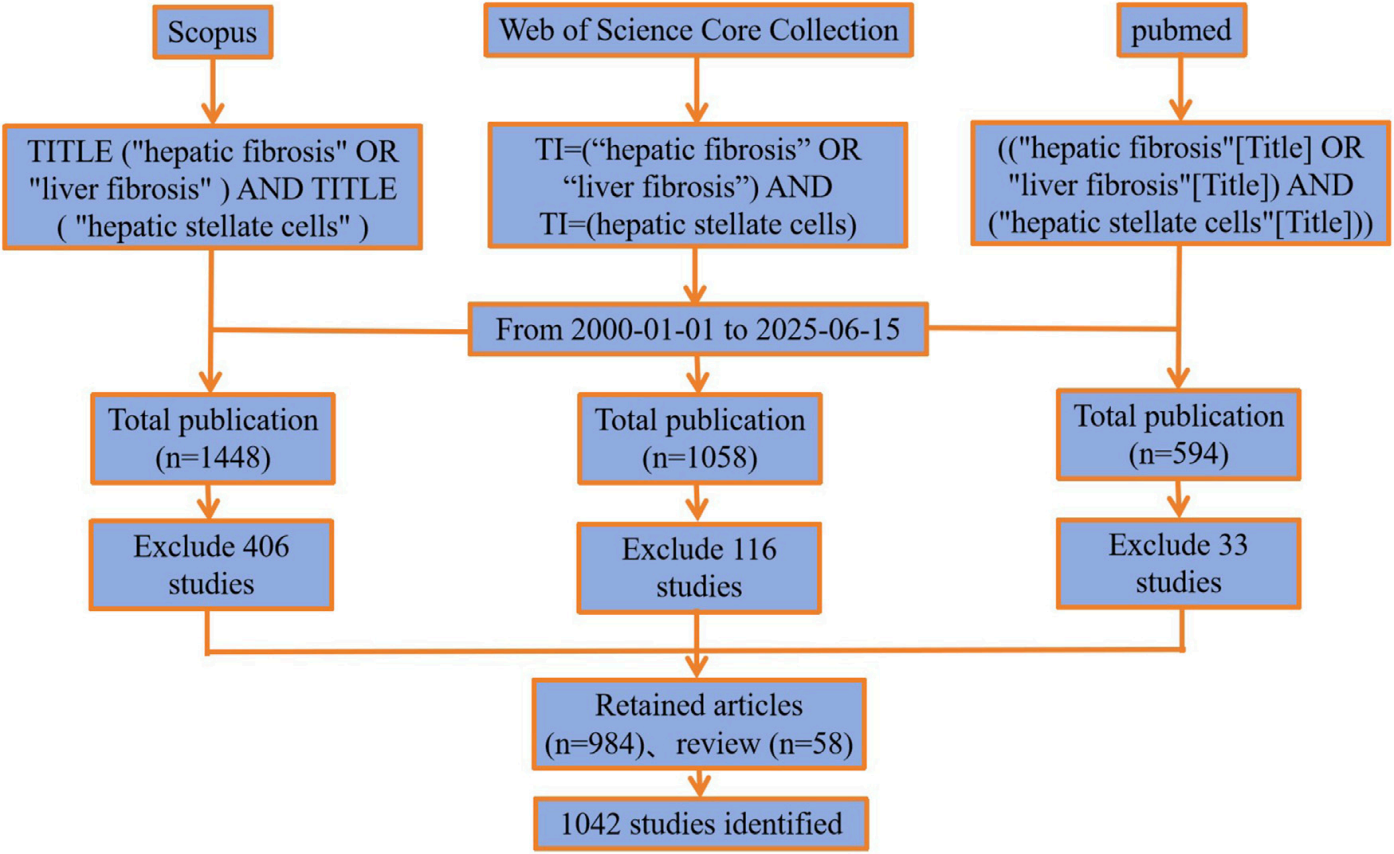
Data collection and retrieval strategy of WoSCC.

Inclusion was limited to records directly relevant to HSCs in LF induction, progression, or reversal, including pharmacological interventions. Exclusions comprised duplicates, non-English abstracts (though full texts were assessed if needed), and irrelevant records (e.g., non-fibrosis contexts). The data collection and inclusion/exclusion flowchart are illustrated in Figure 1.

### 2.4 Data analysis and visualization

From the retrieved articles’ full records and cited references, we extracted information on countries/regions, institutions, authors, journals, keywords, and references for analysis. First, ClinicalTrials.gov was used to retrieve and analyze clinical trial progress, while Innojoy was employed for patent data analysis. This multi-source integration allowed us to assess research trends, hotspots, and translational potential in HSC-targeted LF therapies.

Subsequently, we used CiteSpace 6.2. R4 software, which is compatible with WoSCC literature data and capable of analyzing complex documents (Chen C. et al., 2012). With this tool, we analyzed the intellectual interaction and structural relationships among research components from temporal and spatial perspectives. The results were visualized in network, overlay, and density formats. Different color levels represent clusters and chronological order, whereas lines and circles indicate the degree of research association and subject matter, facilitating for knowledge map mining, analysis, and synthesis.

The parameter settings of CiteSpace 6.2 R4 were as follows: time slicing from 2000 to 2025, with 1 year per slice; data threshold set to top N = 50, and other variables at default values. The pruning method was Pathfinder combined with Pruning sliced networks to simplify the network and highlight essential features.

Mediating centrality (Freeman, 1977) served as a key indicator and “bridge” for assessing the importance of network nodes. Nodes with high centrality are displayed as a “purple circle” signifying their pivotal role in the network, and based on this, we conducted burst analysis on various node types to better highlight the key topic words in our research field. Finally, we summarized and organized the “analysis results” and “citation report.” Microsoft Excel 2010 was used to organize and analyze publications and citation trends, Origin V 9.1 to plot the exported data, and Microsoft PowerPoint 2010 is used to create the literature flowchart (Figure 1).

## 3 Results

### 3.1 Annual publications and trend analysis

**FIGURE 2:**
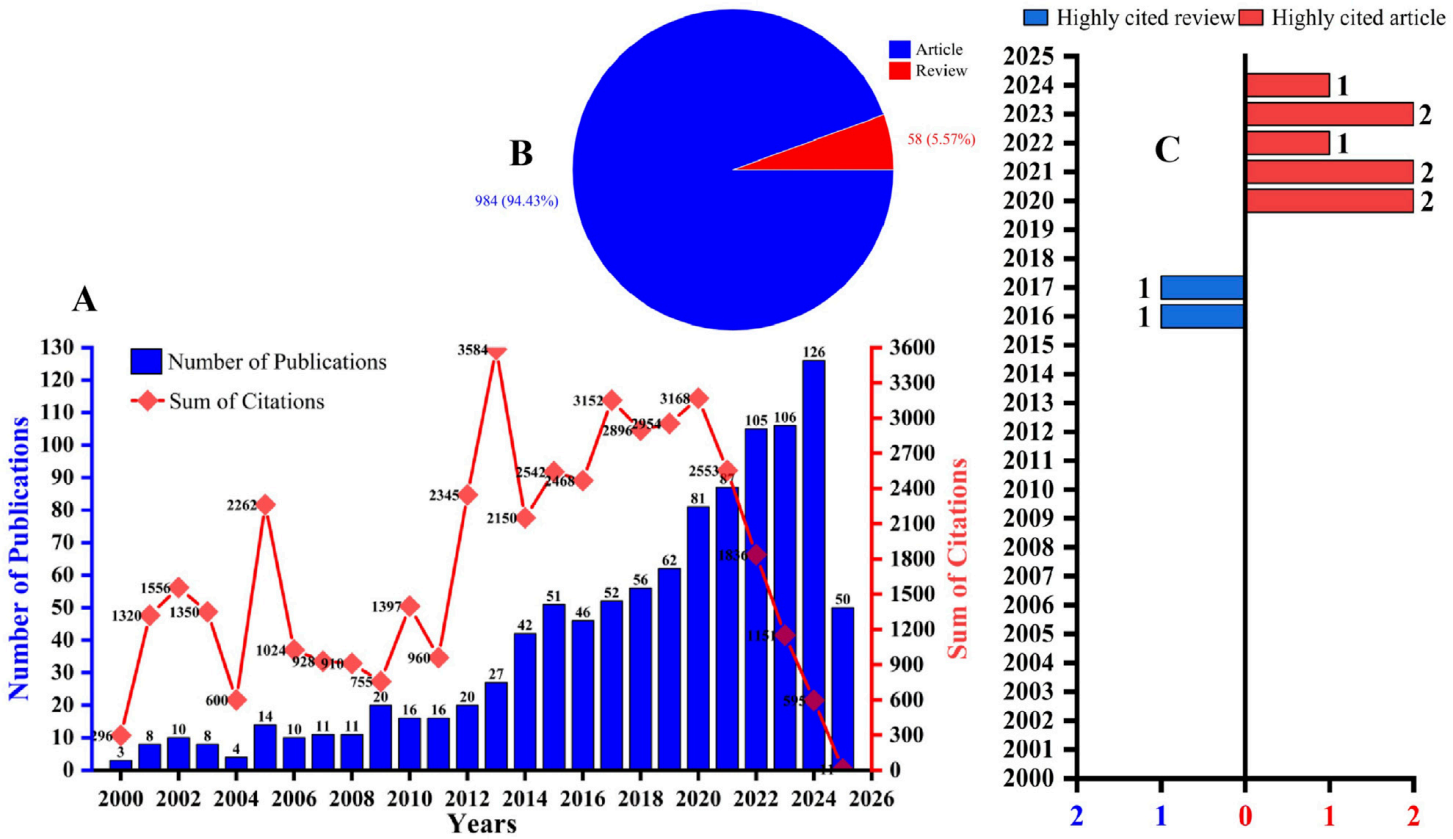
**(A)** Annual trends of publications and citations related to the initiation or improvement of LF by HSCs from 2000 to 2025. **(B)** Types of publications. **(C)** Distribution of review and research articles in highly cited publications.

Records were retrieved from three databases—WoSCC (n = 1,448), Scopus (n = 1,058), and PubMed (n = 594)—restricting the search to English-language articles and reviews on the role of HSCs in LF. Based on these criteria, 1,042 records were retained from WoSCC, 942 from Scopus, and 561 from PubMed. After de-duplication, all records were found to be represented within WoSCC; therefore, subsequent analyses were performed using bibliographic data exported from WoSCC. The WoSCC dataset comprised 984 articles (94.43%) and 58 reviews (5.57%). Of these, 961 items had received citations, totaling 44,763 citations, with a mean of 46.58 citations per cited item. This is illustrated in Figure 2. Figure 2A indicates that the overall number of publications showed a gradual and stable increase with minor fluctuations, indicating that researchers are paying increasing attention to HSCs in the LF process. Figure 2B shows that the proportion of experiments was significantly higher than that of reviews, indicating a high level of scientific research investment in the LF process. Figure 2C shows that the number of highly cited articles gradually increased from 2016 to 2024, and the proportion of experimental articles accounts for 80%, indicating a high experimental interest in HSCs in the LF process. In summary, HSCs can be regarded as a research hotspot and a trend for future LF.

### 3.2 Country distribution

**FIGURE 3:**
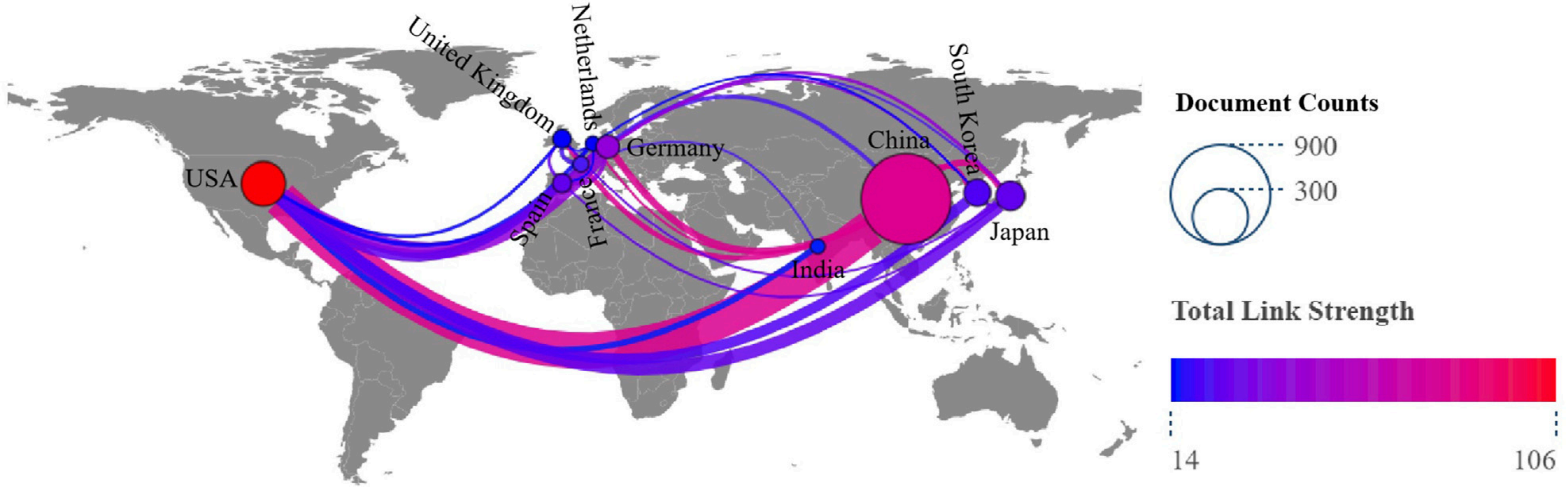
The number of national cooperation networks and publications in the top ten countries for HSCs to induce or improve LF. The size of the nodes in the figure indicates the number of publications, and the line from blue to red indicates that the intensity of cooperation deepens.

The research in this field involves 50 countries. The top ten countries were clustered into different colors based on the proportion of publications and cooperation intensity/centrality. China (672, 0.25) and the United States (162, 0.47) have formed a complex international cooperation network in this field as the main axis (Figure 3). Secondly, the countries with centrality ≥0.1 include the United Kingdom (29, 0.19), the Netherlands (19, 0.18), Germany (44, 0.15), Spain (30, 0.14), France (19, 0.12), and South Korea (60, 0.10). Therefore, an international research system was formed with East Asia, North America, and Europe at its core.

According to the intensity of national cooperation, the United States (8.60) and the United Kingdom (6.47) had extremely high emergence intensities in this field. From 2000 to 2008, researchers mainly conducted studies on restricting and reversing LF by studying the lineage, activation, and apoptosis of HSCs (Xu et al., 2005; Krizhanovsky et al., 2008; Elsharkawy et al., 2005). From 2011 to 2015, the Chinese region mainly researched the inhibition of HSCs activation by studying the effective components of traditional Chinese medicine and traditional Chinese medicine compounds (Liu et al., 2015; Chen JY. et al., 2012), a microcosm of traditional Chinese medicine that conducts LF research at the cellular and pathway levels internationally. In recent years, Iran has committed to researching the prediction and targeted regulation of LF markers (Keshavarz Azizi Raftar et al., 2021), an important follow-up research topic.

### 3.3 Distribution of research institutions

**FIGURE 4:**
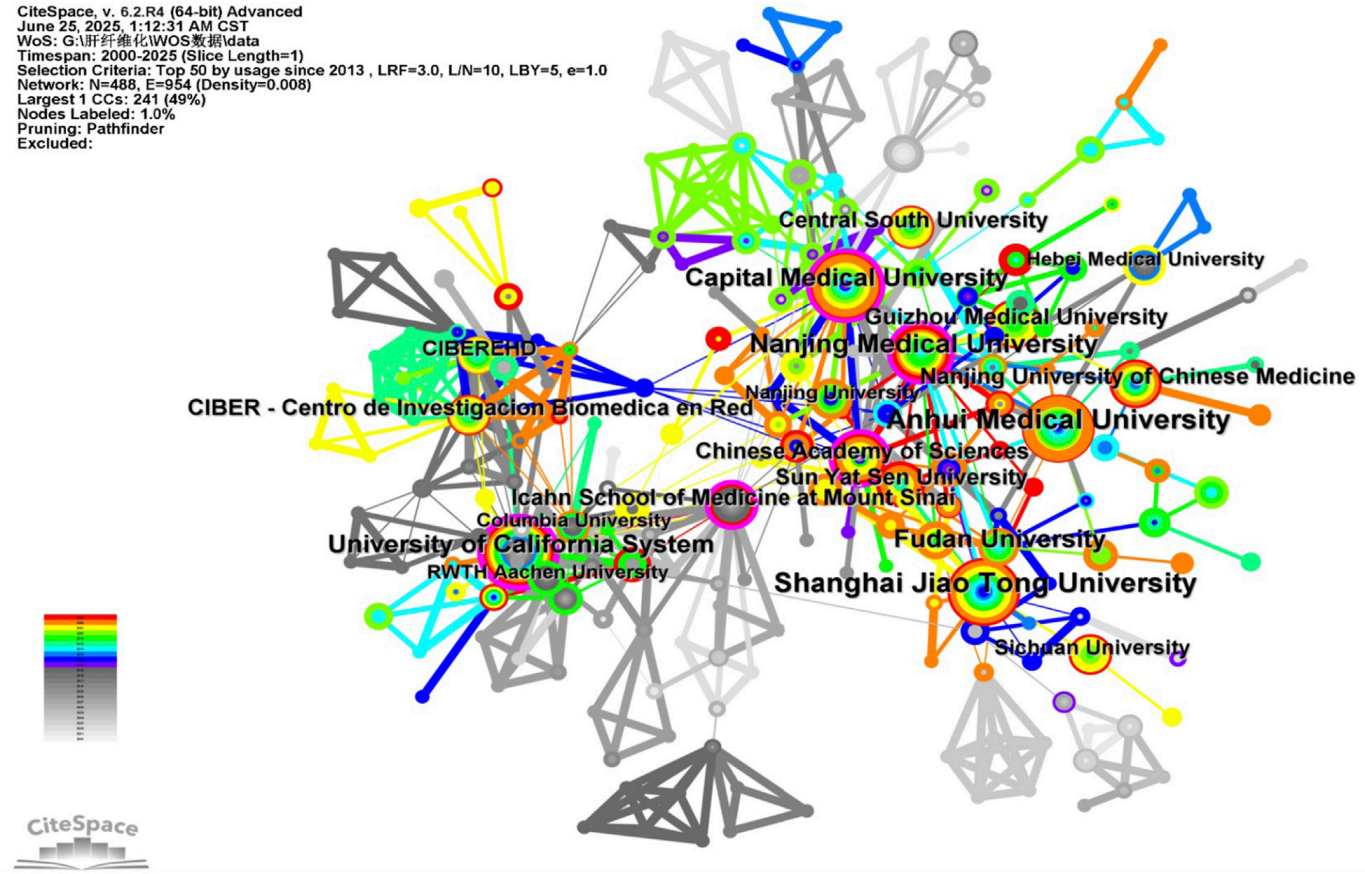
The main research institutions and cooperative relationships in the field of LF caused or improved by HSCs. The node size and color represent the number of publications and the year of publication, respectively, and the purple circle is the core institution.

A total of 488 institutions in this field were dedicated to HSCs and LF research, forming 954 complex cooperative relationships. As shown in Figure 4, the Icahn School of Medicine at Mount Sinai acts as the main connecting hub between the Chinese Academy of Sciences and the University of California, closely linking universities and research institutions between China and the United States and forming important cooperative relationships with CIBEREHD (Spain), RWTH Aachen University (Germany), and other European countries, further promoting the globalization of LF research cooperation.

Among the top ten institutions in terms of publication volume are eight from China and two from the United States, as shown in Supplementary Table S1. Regarding centrality analysis, the top five were the Icahn School of Medicine at Mount Sinai, Nanjing Medical University, the Chinese Academy of Sciences, the University of California, and Capital Medical University. In terms of publication time (centrality ≥0.10), the main institutions include the Icahn School of Medicine at Mount Sinai (2002, 0.18), the Chinese Academy of Sciences (2005, 0.10) and Nanjing Medical University (2013, 0.11). Through burst analysis, it was found that the University of Southampton (6.11), the Icahn School of Medicine at Mount Sinai (5.56), and Nanjing Medical University (4.72) had higher burstiness. Considering these factors, China (Chinese Academy of Sciences, Nanjing Medical University) and the United States (Icahn School of Medicine at Mount Sinai, University of California) can be regarded as hotspots for future research on the LF of HSCs.

### 3.4 Distribution of authors and co-cited authors

**FIGURE 5:**
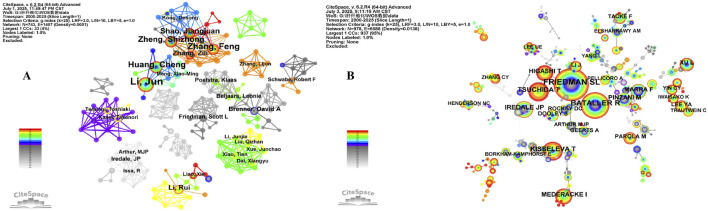
**(A)** The author collaboration network in the field of HSCs triggering or improving LF, with node size and color representing the number of articles published by the authors and the year of publication, respectively. **(B)** Co-cited author cooperation network.

In total, 743 authors contributed to the research on HSCs and LF. The top ten prolific authors published 111 papers, accounting for 10.65% of the total papers, as shown in Supplementary Table S2. Among the top ten authors, eight were from China and two from the United States. Among them, Zhang, Feng from Nanjing University of Chinese Medicine had the highest number of publications (Udomsinprasert et al., 2020), accounting for 1.82% of the total number of publications, followed by Li, Jun from Anhui Medical University (Balmer and Blomhoff, 2002), accounting for 1.72% of the total number of publications. The top ten authors formed six major research networks based on their institutions (Figure 5A). The team led by Zhang Feng from Nanjing University of Chinese Medicine had the highest number of publications, the largest collaborative network, mainly studied the effect of liver-acting blood-activating and blood-stasis-removing traditional Chinese medicines on HSCs (Lu et al., 2016; Sun et al., 2022), with an average citation rate of 43.21. The team led by Li Jun from Anhui Medical University, mainly focusing on the activation/inhibition of HSCs mediated by LEFTY2, DNMT1, and other TGF-β/Smad signaling pathways, thereby revealing the biological process of LF (Yang et al., 2020; Bian et al., 2014), with an average citation rate of 35.28. The team led by Friedman, Scott L from the Icahn School of Medicine at Mount Sinai had the highest average citation rate, mainly studying the activation of HSCs during the LF process mediated by the MPI-induced mannose metabolism pathway (Higashi et al., 2017; DeRossi et al., 2019), with an average citation rate of 378.80. From 2020 to 2023, the emergence intensity was mainly led by the team around Li Rui (4.30) from Sichuan University, who studied the role of Sirt6/CREKA-Lip in HSCs and LF using gene knockout technology and targeted therapy methods (Zhang et al., 2021; Li et al., 2023), with an average citation rate of 36.50. A comprehensive analysis revealed that the research teams led by Zhang, Feng, Li, Jun had a high density around the timeline and were high-yielding points for paper production. Although the teams led by Brenner, David A., Friedman, and Scott L. have continued their research until now, with relatively few publications, their citation rates are extremely high, making them core research teams.

Among the cited authors (Figure 5B), 978 authors and 6588 relationship networks were identified. FRIEDMAN SL., from the Icahn School of Medicine at Mount Sinai in the United States, had the highest citation frequency (543, 0.05), and BATALLER R., from the University of Barcelona in Spain, had the highest centrality (349, 0.10) (Supplementary Table S3). FRIEDMAN SL. elucidated the concept of LF from concept to treatment (Trautwein et al., 2015) and studied the relationship between HSCs and LF from aspects of cell activation, metabolic regulation, etc. (Tsuchida and Friedman, 2017; Trivedi et al., 2021). BATALLER R is mainly engaged in the research of nonalcoholic fatty liver disease diagnosis, mechanism, liver transplantation, etc. (Ma et al., 2022; Rinella et al., 2023). Based on Figure 5B and the emergent analysis of co-cited authors, TSUCHIDA T (19.30) and FRIEDMAN SL (12.86) from the Icahn School of Medicine at Mount Sinai in the United States established a cooperative relationship, and their emergence intensity was extremely high between 2015 and 2023, indicating that they may become a hot research team in this field in the near future.

### 3.5 Distribution of top 15 high-cited articles

The top 15 studies that induced or improved LF in HSCs are listed in Table 1. The top 15 papers were cited 8,144 times, accounting for 18.19% of the total citation rate. These papers were mainly published in top international journals, including four in “Gastroenterology,” two in “Hepatology,” and one in “Gut.” As shown in Table 4, the United States published the most papers (73%), and 2/3 of them were original. The research methods involved multi-institutional and multinational cross-border cooperation. The top five reviews explored the pathology and etiology of HSC activation and targeted treatments for LF. The top ten experimental articles, mainly from “Nat Commun” and “Gut,” first achieved the technological breakthrough of serum-free culture (Xu et al., 2005) and scientifically revealed from the perspective of evidence-based medicine that HSCs are the main contributors and target sources in the process of LF (Mederacke et al., 2013). The survival and fibrosis of HSCs are closely related to inflammatory and growth factors (Hammerich and Tacke, 2023), laying a foundation for subsequent research on the mechanism of LF.

**TABLE 1:** Top 15 cited articles in the field of LF initiated or improved by HSCs.

Rank	DOI	First author	N. of institutions	Journal	IF (2025)	Year	Citations	Type
1	10.1016/j.addr.2017.05.007	Higashi T (United States of America)	2	Adv drug deliv Rev	17.298	2017	1,086	Review
2	10.1038/ncomms3823	Mederacke I(United States of America)	2	Nat commun	15.620	2013	1,076	Article
3	10.1136/gut.2004.042127	Xu L (United States of America)	1	Gut	25.503	2005	846	Article
4	10.1002/cphy.c120035	Puche J E (United States of America)	2	Compr physiol	5.181	2013	606	Review
5	10.1053/j.gastro.2012.05.049	Meng F L (United States of America)	9	Gastroenter-ology	25.214	2012	530	Article
6	10.3748/wjg.v22.i48.10512	Zhang C Y(China)	2	World J gastroenterol	5.443	2016	468	Review
7	10.1002/hep.26429	Pradere J P(United States of America)	7	Hepatology	14.934	2013	462	Article
8	10.1172/JCI200318212	Bataller R (United States of America)	3	J clin invest	13.275	2003	461	Article
9	10.1055/s-2001-17558	Bataller R (United States of America)	1	Semin liver dis	4.257	2001	402	Review
10	10.1053/j.gastro.2012.06.036	Troeger J S(United States of America)	4	Gastroenterol-ogy	25.214	2012	401	Article
11	10.1053/j.gastro.2004.08.001	Fiorucci S(Italy)	2	Gastroenterol-ogy	25.214	2004	394	Article
12	10.2741/reeves	Reeves H L (United States of America)	1	Front biosci	2.953	2002	379	Review
13	10.1074/jbc.M111490200	Murphy F R (United Kingdom)	2	J biol chem	3.817	2002	371	Article
14	10.1002/hep.25744	Kong X N(United States of America)	3	Hepatology	14.934	2012	347	Article
15	10.1016/S0016-5085 (03)00666-8	Dooley S(Germany)	2	Gastroenterol-ogy	25.214	2003	315	Article

### 3.6 Keywords analysis

Analysis of keyword co-occurrence is the main path to understanding the research topics and scope of HSCs in the LF field. Therefore, we listed the high-frequency keywords with frequency ≥20, as shown in Supplementary Table S4. Based on frequency, keywords with frequency ≥100 mainly focus on “liver fibrosis” and “hepatic stellate cells,” exploring the impact of “tgf beta” signaling pathway on gene and RNA “expression” in HSCs “activation,” “proliferation,” “apoptosis” and other processes; based on centrality, keywords with frequency ≥20 mainly focus on HSCs “activation” and “inhibition,” studying “extracellular matrix,” “TGF-β,” “receptor,” “expression,” “apoptosis” and “cirrhosis” through “*in vivo*” experiments. In summary, “mechanisms” and “expression” run through the beginning and end of HSCs and LF research.

**FIGURE 6:**
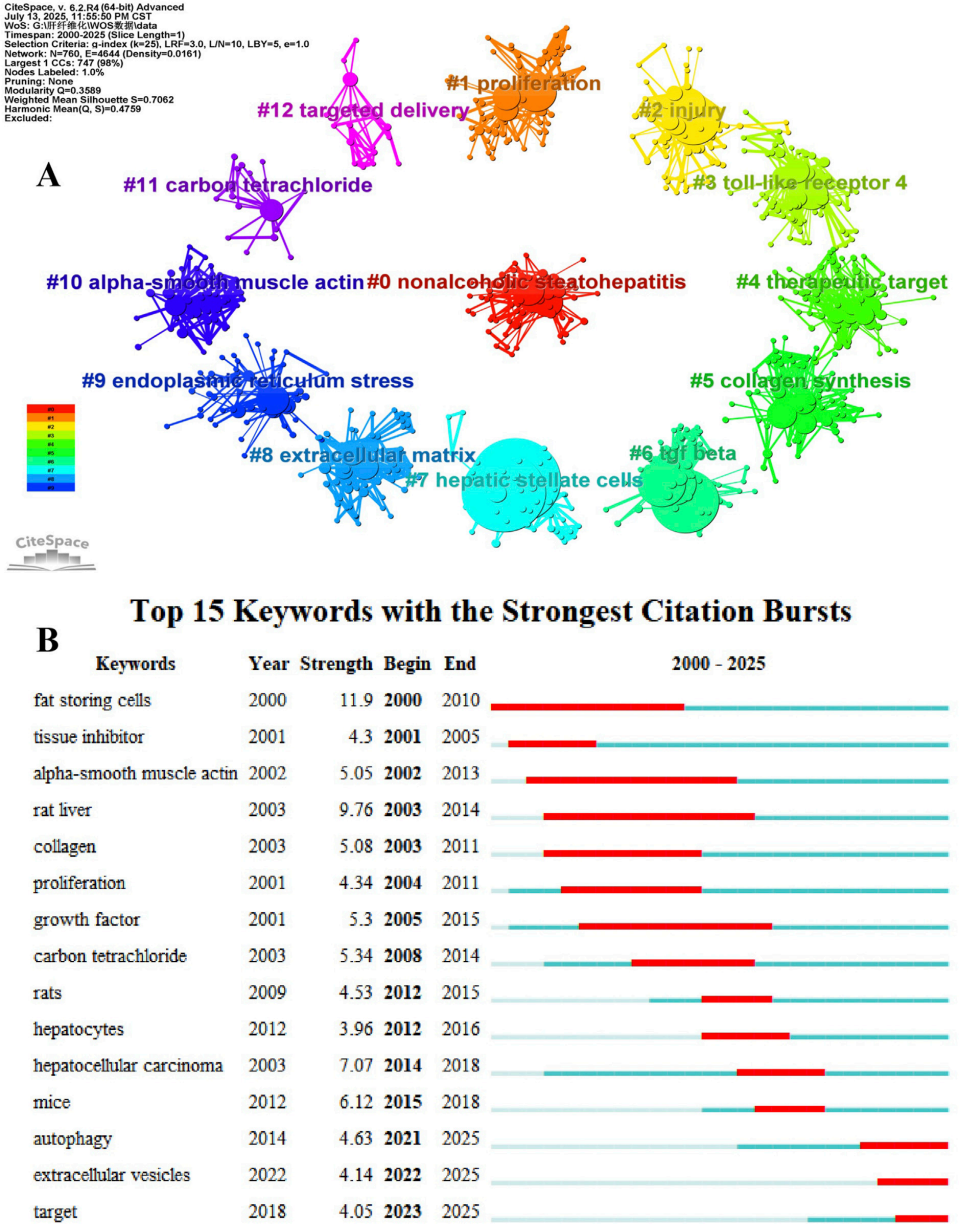
**(A)** The main clustering networks in the field of HSCs inducing or improving LF have a total of 13 valid clusters, and the cluster colors distinguish the corresponding clustering groups and labels. **(B)** The top 15 keywords in terms of emergence intensity, with red lines indicating the emergence year intervals.

To understand the latest research progress on HSCs in the LF process, we clustered the collinear keywords and obtained 13 valid clusters (Figure 6A). According to the main research content, the 13 valid clusters were focused on: fibrosis etiology and causes (#0, #7, #11), fibrosis molecular mechanisms (activation: #1, #5, #6, #8, #10; inhibition: #2, #3, #9), fibrosis treatment (#4, #12), and three other aspects. According to the average year of the emergence of valid clusters, it was found that from 2005 to 2018, the mechanism of HSCs activation preceded inhibition. Targeted drug delivery and treatment are currently the hotspots and frontiers of research based on the etiology of fibrosis and degree of injury.

The keyword burst analysis identified 15 burst terms (Figure 6B) that described hotspots and future research trends in LF research over a certain period. Based on the burst strength, the top five burst terms were “fat storing cells,” “rat liver,” “hepatocellular carcinoma,” “mice,” and “carbon tetrachloride,” indicating that exploring the molecular mechanisms of rats and mice as *in vivo* research objects is the current mainstream trend.

### 3.7 Medical patent analysis

**FIGURE 7:**
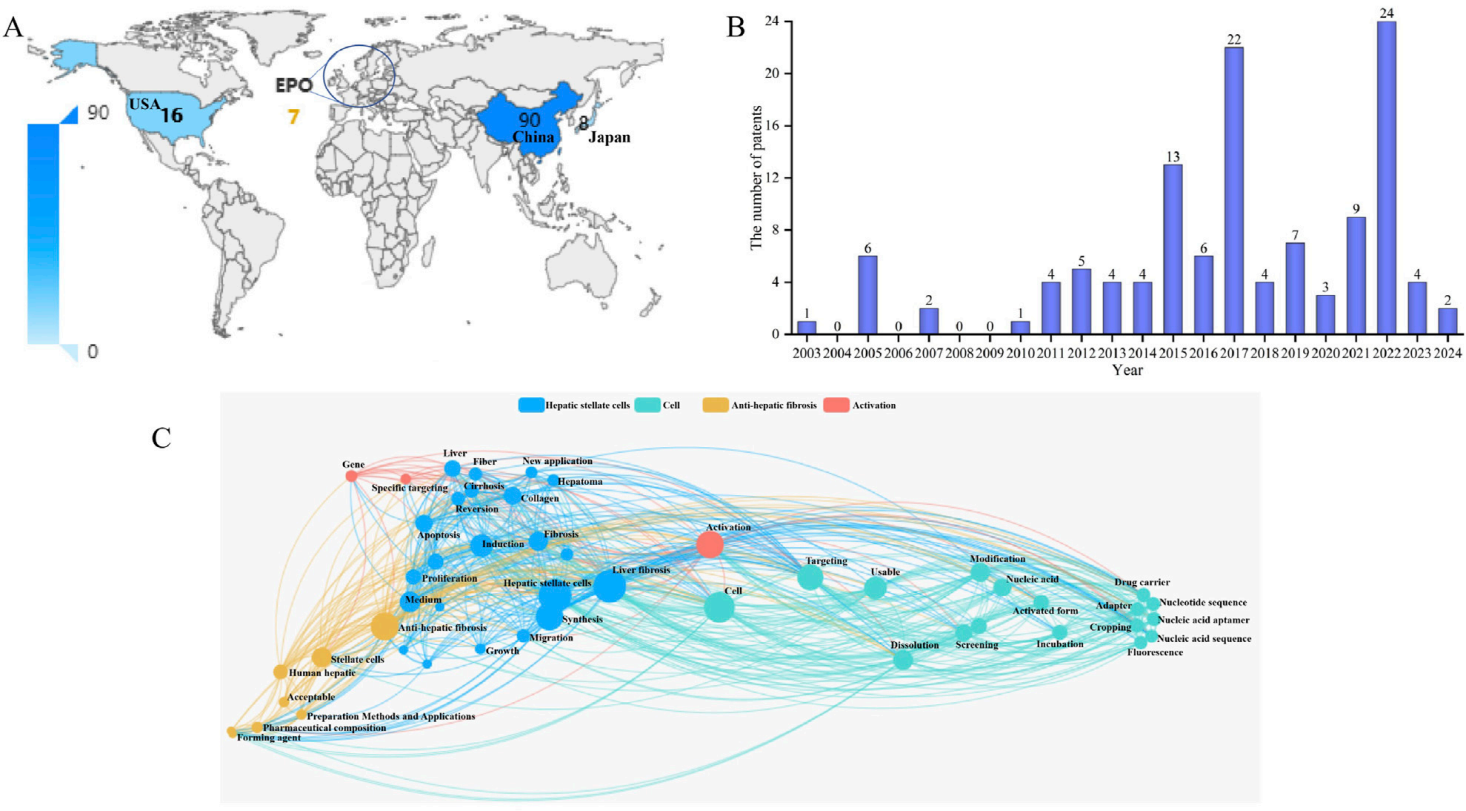
**(A)** World map and number of patents in major countries. **(B)** The annual number of highly relevant patents. **(C)** Patent keyword cluster analysis, with colors representing different types of patents.

China (90 patents) emerged as the most densely populated region for global patent filings, potentially linked to the high domestic demand due to prevalent liver diseases and supportive research policies, such as those promoting traditional Chinese medicine for anti-LF studies. This was followed by the United States (United States, 16 patents), Japan (8 patents), and the European Patent Office (EPO, 7 patents) (Figure 7A).

Patent development stage (Figure 7B), the initial phase (2003–2014) featured persistently low patent volumes (≤6 per year), reflecting insufficient understanding of HSC-targeted mechanisms, immature translational technologies, and consequent limitations in patent output. The growth phase (2015–2020) showed notable peaks in 2016 (13 patents) and 2017 (22 patents), suggesting associations with the infiltration of single-cell sequencing and gene-editing technologies (e.g., CRISPR), or surges in anti-fibrotic drug development (e.g., targeting TGF-β and PDGFR pathways), which propelled basic research toward patent conversions. The fluctuating development phase (2021–2024) reached a new high in 2022 (24 patents) before declining, indicating the field’s entry into a stage of technological iteration and differentiation—characterized by patent bursts driven by novel targets and strategies (e.g., exosome-based targeting and AI-assisted drug design), alongside slowdowns in traditional directions due to clinical bottlenecks.

Patent keyword cluster analysis (Figure 7C), Cluster (blue): Centered on “hepatic stellate cells,” with high-frequency nodes such as “liver,” “activation,” “apoptosis,” and “proliferation” dominating. This reflects a research focus on HSC biological characteristics, including activation, apoptosis, and phenotypic transformation, which are core drivers of LF pathogenesis. Anti-fibrotic therapy cluster (orange): Key nodes including “anti-liver fibrosis,” “phytomedicine,” and “drug delivery” highlight therapeutic strategies targeting HSCs. Natural products and novel delivery systems emerge as important directions for anti-fibrotic interventions. Cellular and molecular mechanism cluster (cyan): Encompassing nodes like “cells,” “screening,” “modification,” and “fluorescence,” emphasizing techniques such as cell screening, gene modification, and imaging to support mechanistic studies and therapeutic development. Activation and regulation cluster (red): Revolving around “activation,” “signaling pathways,” and “inflammation,” focusing on molecular cascades regulating HSC activation, which represent critical links in fibrosis progression.

Based on a unique categorization of primary technological innovations from 121 patents (Table 2), the development of novel active ingredients constitutes the largest technical direction, accounting for 64.5% (78/121) of the analyzed patents. This includes natural product extracts (e.g., Tibetan rhubarb extract CN102225097A), synthetic small molecules (e.g., pyrazolopyrimidine derivatives EP1888074A1), and biomacromolecules such as monoclonal antibodies (e.g., HAb18G/CD147 monoclonal antibody CN101054416A). The second most significant innovation area is delivery systems and formulation technologies, comprising 18.2% (22/121) of the patents. This trend underscores the growing importance of enhancing drug efficacy and specificity through advanced nanotechnology (e.g., oxidized matrine nanoparticles CN102961360A and hyaluronic acid nanomicelles CN107854431A). Emerging therapeutic strategies, including gene therapy (e.g., RNA interference CN105624162A and CRISPR-Cas9 system CN108251423A) and cell therapy, as well as the development of novel disease models and diagnostic tools (e.g., 3D models CN109337860A and targeting peptides CN106699848A), although currently represented by fewer patents, signify the cutting-edge frontiers in this field.

**TABLE 2:** Unique categorization of primary technological innovations in 121 analyzed patents for LF therapies.

Technology field	Core strategy	Number of patents	Representative patent (publication number)
Novel active ingredients	Natural product extracts, chemically synthesized small molecules, biological macromolecules (antibodies, peptides)	78	CN102225097A (extract of Rheum tanguticum), EP1888074A1 (pyrazolopyrimidine derivatives), CN101054416A (HAb18G/CD147 monoclonal antibody)
Delivery systems and formulation technologies	Targeted nanoparticles, liposomes, micelles, cellular preparations, and other novel delivery technologies	22	CN102961360A (oxidized matrine nanoparticles), CN107854431A (hyaluronic acid nanomicelles), CN115322946A (cellular preparation)
Therapeutic methods and mechanisms	Gene therapy (RNAi, CRISPR), cell therapy, induction of apoptosis, inhibition of activation, and other novel mechanisms	15	CN105624162A (siRNA), CN108251423A (CRISPRa), US20140086984A1 (combination therapy)
Models and diagnostic tools	*In vitro* 3D models, organoid models, specific diagnostic molecules (aptamers, peptides)	6	CN109337860A (3D model), CN109880791A (organoid), CN106699848A (targeted peptide)

Analysis of the distribution of patents by IPC codes revealed that the total number of IPC codes (N = 230) exceeded the number of patents, as a single patent may be assigned multiple codes. As shown in Table 3, the vast majority of patents (71.9%, 87/121) were classified under A61P 1/16 (medicinal preparations for treating liver or gallbladder disorders), reinforcing the primary therapeutic objective of these inventions.

**TABLE 3:** Distribution of international patent classification (IPC) codes in 121 analyzed patents for anti-fibrotic therapies targeting liver disorders.

Main IPC code	Technical field description	Frequency of occurrence	Percentage (%)
A61P 1/16	Drugs for treating liver or gallbladder diseases	87	71.9
A61K 31/	Medicinal preparations containing organic active ingredients	65	53.7
A61K 45/06	Mixtures without active ingredients, e.g., chemical cocktail therapy	11	9.1
C12N 15/113	Nucleic acids mediating gene regulation, e.g., siRNA, shRNA	7	5.8
C07K 16/18	Antibodies against bioactive substances or cell surface markers	7	5.8
A61K 9/00	Medicinal preparations characterized by special physical form	6	5
A61K 35/28	Materials from mammals or birds, e.g., stem cells, liver cellsMaterials from mammals or birds, e.g., stem cells, liver cells	5	4.1
C12N 5/071	Undifferentiated human, animal or plant cells, e.g., stem cells or hepatocytes	5	4.1
Others	Includes C07D, A61K47, G01N33, C12Q1, etc.	37	30.6

Furthermore, 53.7% (65/121) of the patents were concurrently classified under A61K 31/(medicinal preparations containing organic active ingredients), which aligns with the prevalence of novel small-molecule compounds. The notable presence of codes such as C12N 15/113 (nucleic acids mediating gene regulation, e.g., siRNA), C07K 16/18 (antibodies), and A61K 9/00 (medicinal preparations characterized by special physical form) highlights the integration of diverse technological approaches—from biotherapeutics to advanced drug delivery systems—in the pursuit of effective anti-fibrotic therapies.

### 3.8 Clinical trial analysis

**FIGURE 8:**
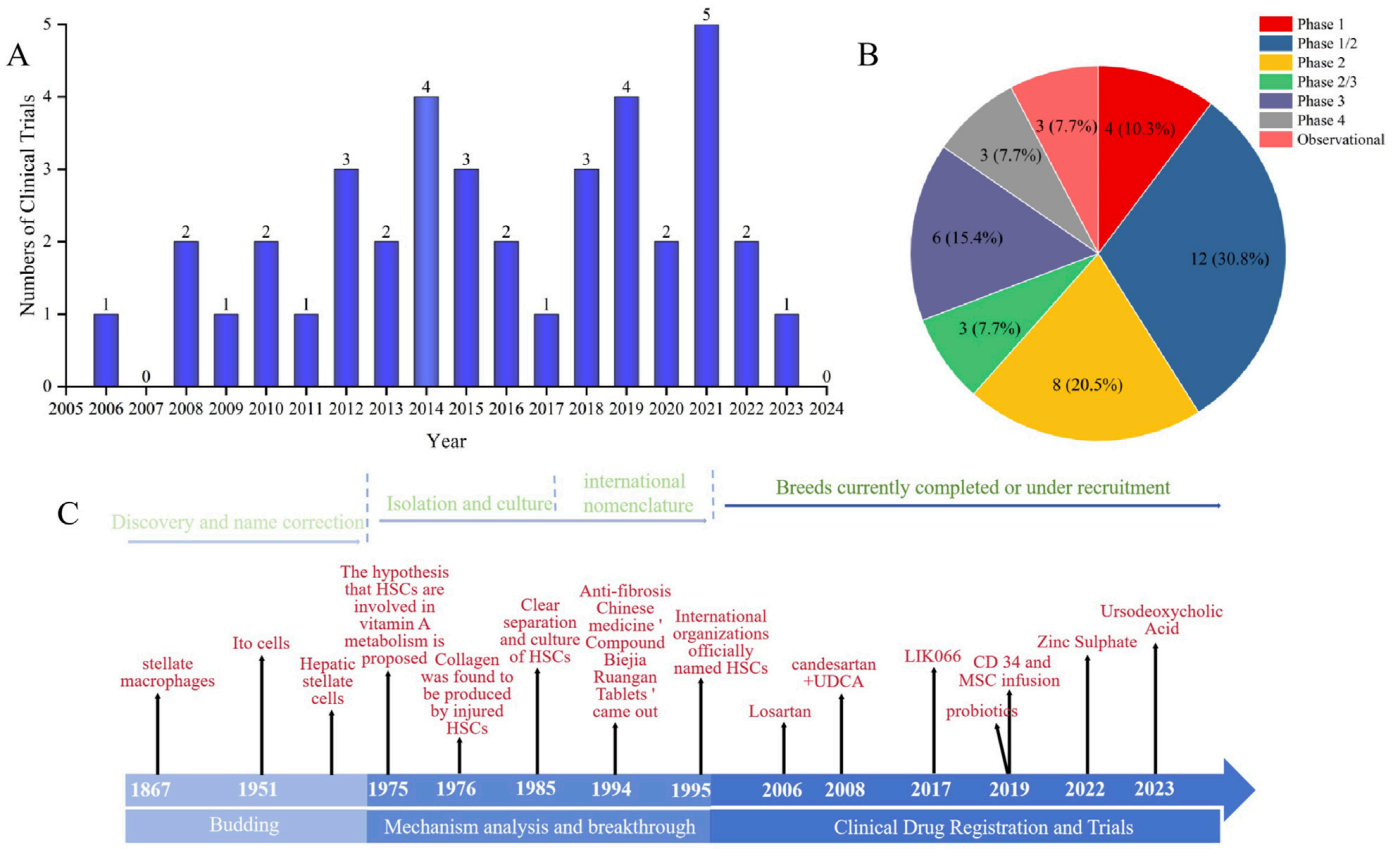
**(A)** annual clinical trial registration dynamics in anti-fibrotic therapies (2005–2024). **(B)** Evolution of clinical trials for HSC-targeted drug interventions (2000 – Present). **(C)** Historical timeline of HSCs discovery and nomenclature (1876–1996).

We have compiled a chronology of landmark discoveries and nomenclature for HSCs and anti-fibrotic therapies since 1876. This timeline highlights pivotal conceptual and research milestones, offering crucial historical context for understanding the progression of HSCs research and drug development in LF (Figure 8C). Since 1876, German scholar Carl von Kupffer (Wake, 2004) observed stellate cells around hepatic sinusoids, which underwent nearly a century of development and nomenclature refinement through the works of Toshio Ito (Ito and Nemoto, 1952) and Kenjiro Wake (Wake, 1971), ultimately bringing HSCs into the view of subsequent researchers. In 1975, Reiner Bauer proposed that Ito cells may participate in vitamin A metabolism and fibrosis, which was later validated by Kent G (Kent et al., 1976) and Scott L. Friedman (Friedman et al., 1985), who isolated and cultured HSCs and demonstrated them as the primary collagen-producing cells in the liver. With the advent of the first anti-fibrotic traditional Chinese medicine “Compound Biejia Ruangan Tablet” in 1994 (Yang et al., 1994), the international community formally named HSCs in 1995 (Shang et al., 2018) (Figure 8C), ushering in an era of clinical research trials for pharmacological interventions.

The exploratory initiation phase (2005–2013) featured annual patent registrations that remained consistently low at 1–3 items, reflecting the field’s early stage of clinical translation. This was followed by a breakthrough growth phase (2014–2021), with notable peaks in 2014 (4 items), 2019 (4 items), and 2021 (5 items), indicating accelerated technical translation toward clinical applications. The fluctuation adjustment phase (2022–2024) showed a decline to 1–2 items per year after 2022, entering a stage of core challenges in clinical translation (Figure 8A).

Among the 39 drug intervention trials, approximately 70% were completed, 15% terminated, and the remainder classified as unknown or not yet recruiting. Geographically, the distribution was dominated by Asia (India, China, Iran) and North America, reflecting regional liver disease burdens. Trial phases included Phase 1/2 (61%), Phase 2/3 (8%), Phase 3/4 (23%) and observational studies (8%), with a median sample size of 50 participants (range: 5–200), most being randomized controlled trials (RCTs) (Figure 8B). In the early period (2000–2010): 6 trials (e.g., NCT00298714 [2003], NCT00990639 [2005]) focused on single small-molecule drugs, such as RAS inhibitors (losartan/candesartan, accounting for 50% of early trials). These targeted the ANG II pathway in HSCs to reduce collagen synthesis, reflecting the prevalence of HCV at the time (40% of early trials were HCV-related). In the mid-period (2011–2019): 17 trials (e.g., NCT03205150 [2017], NCT04243681 [2019], NCT03863730 [2019]) shifted toward cell therapies and biologics (e.g., CD34 and MSC infusions), emphasizing HSC inhibition and liver regeneration. In the recent period (2020 onward): 16 trials (e.g., NCT05465434 [2022], NCT06918080 [2023]) demonstrated diversification, including zinc supplementation, deoxycholic acid, and dietary adjunct therapies. Future trends are expected to transition from single-drug approaches to combination/personalized therapies.

## 4 Discussion

### 4.1 Global trends of HSCs in the study of LF

Using WoSCC as the data source, this study employed CiteSpace 6.2. R4 software to generate a knowledge map of HSCs research in the field of LF from 2000 to 2025, including trends in publication, countries, institutions, authors, highly cited articles, and keywords. This study provides new insights for clinical trials.

Although HSCs research in the field of LF has fluctuated slightly over the past 26 years, it has shown an overall increasing trend. Over the past 5 years, research has accounted for nearly half of the overall research, indicating that HSCs research in the field of LF has entered a rapid development stage. According to the statistics, the included literature is mainly medical and covers eight major fields: hepatology, gastroenterology, pharmacology, cell biology, molecular biology, immunology, toxicology, and chemistry. A total of 1,721 authors from 488 research institutions in 50 countries participated in the relevant research on this topic. China, United States, Japan, and South Korea have a relatively high number of publications. Chinese and American research teams have collaborated closely with institutions in Japan, South Korea, Germany, and other countries, regardless of geographical restrictions. Their contributions and research papers are significant, indicating that research in this field has attracted global attention.

### 4.2 The etiology of inducing HSCs in the process of LF

**FIGURE 9:**
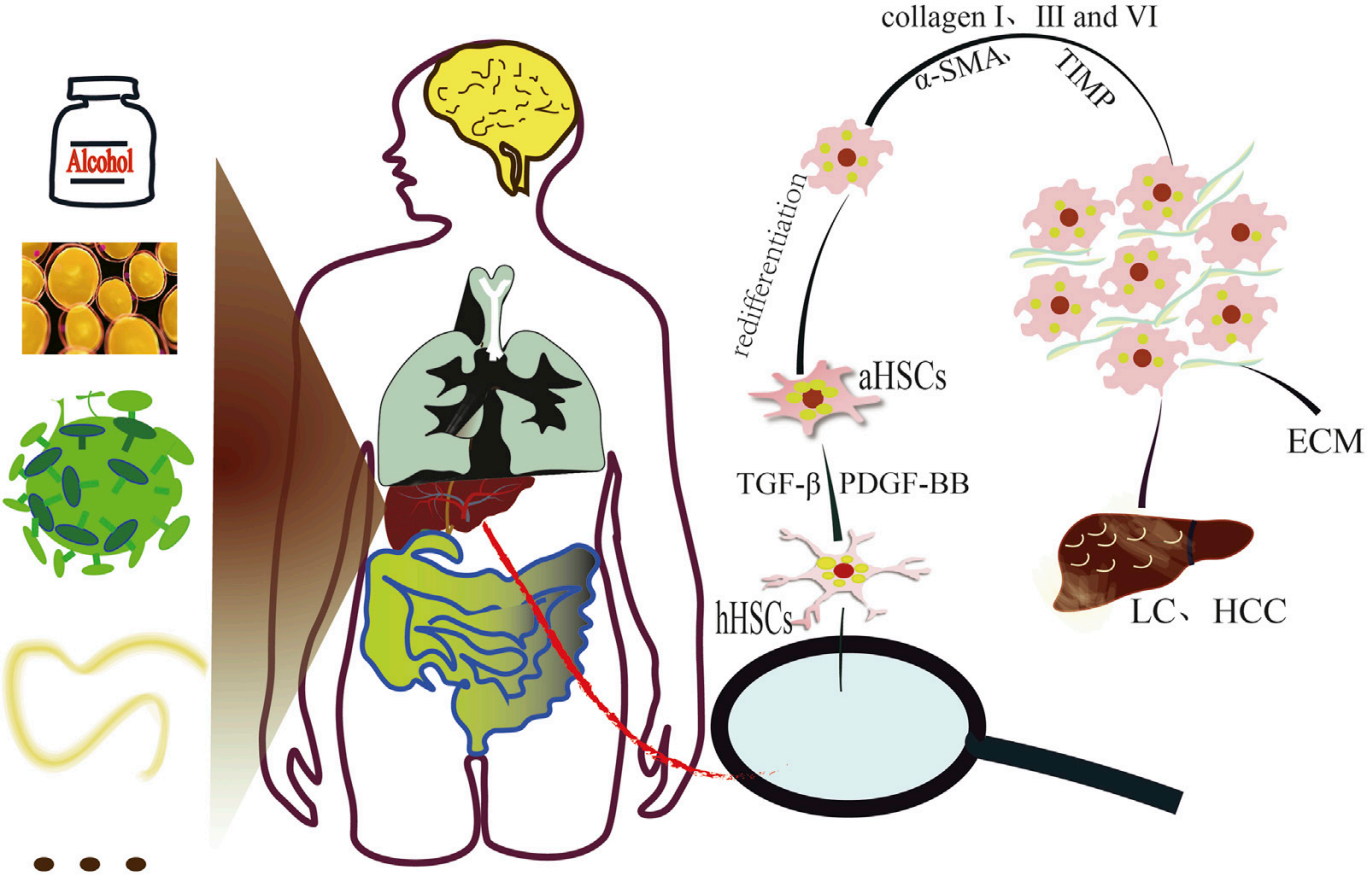
Schematic diagram of the pathogenic factors inducing LF and the pathological activation of HSCs leading to LF.

HSCs are distributed in the Disse’s space around the hepatic sinusoids throughout the liver and are closely associated with chronic liver disease (Araújo Júnior et al., 2016; Li X. et al., 2017). LF induced by alcoholic or NASH, obese steatohepatitis, viral hepatitis (types B and C), autoimmune hepatitis, and metabolic disorders is a typical result of chronic liver injury. Studies have found that CCl_4_, biliary ligation, and fatty liver disease can indirectly or continuously cause liver damage, activating quiescent HSCs with various fibrotic mediators such as TGF-β and PDGF-BB (Kim et al., 2022; van Dijk et al., 2020) to transform into contractile, proinflammatory, and fibrogenic myofibroblasts. This process leads to increased deposition of ECM (Matsuda et al., 2018), resulting in scar formation and gradual liver function failure, ultimately leading to LC, HCC, and death (Figure 9).

### 4.3 Pathological mechanism

#### 4.3.1 Alcohol

Excessive alcohol consumption leads to steatohepatitis, which further develops into alcoholic liver disease. After alcohol is absorbed by the HSCs, ADH (alcohol dehydrogenase) and ALDH (aldehyde dehydrogenase) are activated (Casini et al., 1998). Ethanol molecules are converted to CO_2_ and H_2_O through metabolic reactions. However, in this process, ADH catalyzes the oxidation of retinol in HSCs, resulting in the loss of retinol and activation of HSCs. In addition, alcohol regulates the Nrf2-Keap1-ARE pathway in HSCs to induce autophagy-regulated oxidative stress (Xie et al., 2018) and at the same time, activates CD73 to regulate the AMPK/AKT/mTOR signaling pathway to promote autophagy and activation of HSCs (Wu et al., 2022), thereby increasing the expression levels of alanine aminotransferase and aspartate aminotransferase in the serum, increasing the deposition of α-SMA and type I collagen, and worsening LF.

#### 4.3.2 NASH

The metabolic syndrome usually causes NASH and is closely associated with obesity, insulin resistance, and dyslipidemia. Serum obesity-related factors (leptin, IL-6) increase the expression of lipoprotein lipase (LPL) in HSCs through STAT3 signaling pathway conduction, which leads to an increase in intracellular free cholesterol content and promotes TLR4 signaling and inhibits the expression of *Bambi*, stimulates TGF-β induction of HSCs, and accelerates the production of intracellular collagen (Teratani et al., 2019). Similarly, a lack of *Angptl4* can cause LPL-specific expression, deepening the degree of LF (Teratani et al., 2021). Insulin participates in the serum pathway to stimulate the expression of α-SMA in quiescent HSCs by mediating the PI3K/Akt-p70S6K pathway (Cai et al., 2017), promoting HSCs proliferation and fibrosis development.

#### 4.3.3 Viral infection

Infection with hepatitis viruses (both Hepatitis B and C) can induce liver inflammation. Hepatitis B virus e antigen (HBeAg), as one of the pathogenic factors of Hepatitis B, can directly induce the activation of HSCs by autocrine TGF-β (Zan et al., 2013). However, another Hepatitis B virus antigen, Hepatitis B virus x antigen (HBxAg) (Zhang et al., 2020), can promote the recruitment of helper T cell 17 (Th17 cells) in the liver, which secrete inflammatory factors, such as IL-22 and IL-17A, thus accelerating the phosphorylation and activation of HSCs by AKT and PI3K and the secretion of fibrotic products. The Hepatitis C virus mainly infects hepatocytes and secretes exosomes carrying miR-19a, which are internalized into HSCs and downregulate the expression of SOCS3, promoting STAT3 phosphorylation and mediating the activation of HSCs by TGF-β to secrete connective tissue growth factor (CTGF) (Devhare et al., 2017), stimulating fibroblast proliferation and collagen deposition. Additionally, Hepatitis C core proteins (Wu et al., 2013; Shahin et al., 2020) can induce obesity receptors and inflammatory responses to participate in the activation of HSCs and the occurrence of fibrosis through lipid metabolism pathways.

#### 4.3.4 Schistosome infection

The liver is one of the most common organs parasitized or invaded by parasites, with the main characteristic being that worms or worm eggs can survive for a long time in the host body and cause granulomas in the parasitized area. Then, the soluble antigens (SEA) released by the eggs can cause granuloma inflammation, inducing the immune system to shift from Th1 to Th2 response and continuously stimulating HSCs to produce NLRP3 inflammatory body activation of intracellular caspase-1 and TGF-β expression. The activation of HSCs can regulate the recruitment of macrophages and other immune cells (such as neutrophils, monocytes, and eosinophils) into the granuloma to mediate their response (Carson et al., 2018). Subsequently, with the death and continuous excretion of captured eggs, fibrotic plaques remaining in the liver eventually develop into LF and LC.

### 4.4 Endoplasmic reticulum (ER) stress

HSCs activation, proliferation, and the excessive secretion of the ECM are closely related to protein folding and calcium homeostasis in the ER. The ER can effectively reverse LF by adapting, protecting, and initiating programmed cell death triggered by many physiological and pathological factors (Carson et al., 2018; Folch-Puy et al., 2016). Protein kinase R-like ER kinase is a sensor of ER stress and an important branch of the unfolded protein response (UPR). It mediates the phosphorylation of HNRNPA1 Thr51 to inhibit the catalytic cleavage of primary-MIR18A by HNRNPA1, leading to the overexpression of Smad2 in HSCs (Koo et al., 2016). At the same time, the activation of another branch of UPR is regulated by the ER stress sensor inositol-requiring enzyme 1α (IRE1α) (Liu et al., 2019), which can induce the phosphorylation of IRE1α downstream of SMAD2/3 by TGF-β, thereby activating the ASK1-JNK signaling cascade and promoting the activation and expression of downstream c/EBPβ-p300, exacerbating the excessive accumulation of the ECM. Calcium calmodulin-dependent protein kinase II (CaMK II) can regulate calcium homeostasis in the HSCs. After TGF-β stimulates HSCs, the activity of CaMK II is inhibited, the intracellular Ca^2+^ level and the expression of GRP78 are increased, *Bcl-2* is inhibited, and the apoptotic proteins Caspase-12 and Bax are activated and expressed, which increases the apoptosis of activated HSCs (Liu et al., 2021).

### 4.5 TGF-β/SMAD signal transduction

**FIGURE 10:**
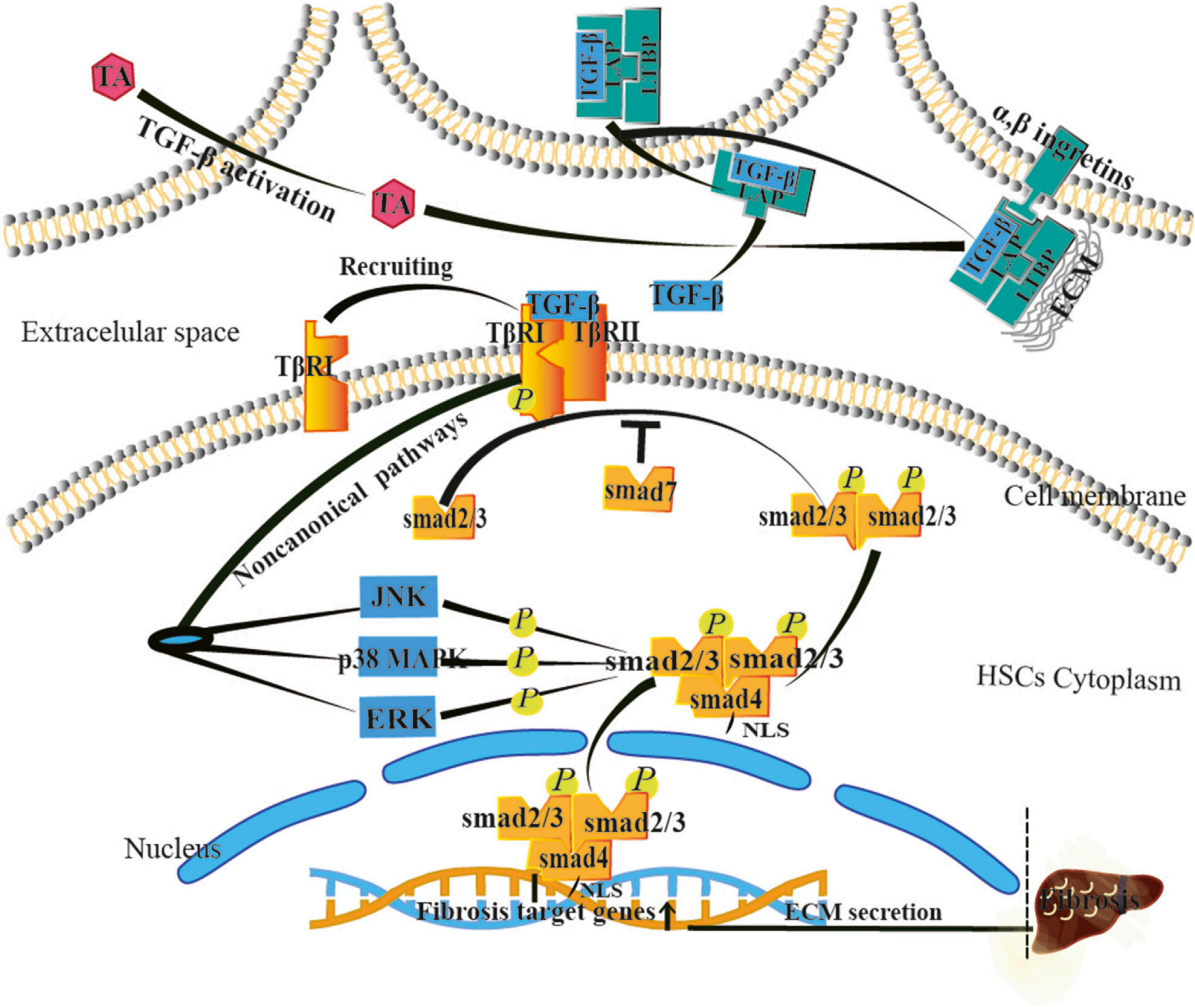
Research on the TGF-β/SMAD or non-SMAD signaling pathways in the process of LF.

The liver microenvironment is the main location where HSCs receive external information and survive and has a high degree of dynamics during liver injury. Corresponding information transmission changes between microenvironment secretory factors and HSCs accompany different stages of LF. The key factor affecting this physiological/pathological change is TGF-β, mainly from HSCs, liver endothelial cells, macrophages, and hepatocytes (Ni et al., 2017; Krenkel and Tacke, 2017). When the liver is stimulated by alcohol, obesity, virus, and schistosomiasis hepatitis, furin-like protease in liver immune cells cleaves and activates TGF-β molecules (Shi et al., 2011), allowing the C-terminal of TGF-β molecules to bind to the N-terminal of Latency associated protein (LAP) to form latent TGF-β complexes. During the excretion process, they need to bind to Latent TGF-β binding protein (LTBP) (Robertson and Rifkin, 2016) and then be excreted outside the cell and enter the ECM (Figure 10). Integrin and TGF-β activator (TA) can induce conformational changes in LAP, successfully binding TGF-β to HSCs (Khan and Marshall, 2016). TGF-β is a major pro-fibrotic cytokine that binds to the TGF-β type II receptor (TGF-βRII) on the surface of HSCs and recruits the TGF-β type I receptor (TGF-βRI) to change its conformation and acquire kinase activity. It then phosphorylates downstream Smad2/3 proteins and binds to Smad4 to form a functional heterodimer complex (Carthy, 2018; Derynck and Budi, 2019), exposing the nuclear localization sequence (NLS) to enter the nucleus and bind specific DNA sequences to activate fibrosis-targeted gene-mediated fibrotic reactions (Yoshida and Matsuzaki, 2012). In addition to the classical Smad pathway, TGF-β receptors can also directly interact with non-canonical signaling transduction such as Jun N-terminal kinase (JNK), p38 MAPK, and extracellular signal-regulated kinase (ERK) (Matsuzaki, 2009) (Figure 10), thereby initiating parallel Smad-independent signaling transduction for corresponding effects. In addition to the activation pathways mentioned previously, inhibitory Smad7 has a negative feedback protective effect during fibrosis (Feng et al., 2015).

### 4.6 Experimental model

Studying experimental animal models that simulate the occurrence and development of human diseases is an effective way to propose new theoretical hypotheses and solve major clinical problems. Currently, the commonly used experimental animals for establishing LF models include rats, mice, and rabbits. During the experiment, modeling was performed using chemical, dietary, surgical, genetic, and other means. Standard modeling methods are shown in Table 4, depending on the different etiologies of LF in clinical practice.

**TABLE 4:** Animal models of LF.

Modeling method	Inducing drug/Modified gene	Method	Dosage	Modeling time	Advantages	Disadvantages	References
Chemical injury model	NDMA	Intraperitoneal injection	0.5% NDMA,2 mL/kg	3–4 weeks	Stable model with high success rate	Expensive and has strong hepatotoxicity	Zhang et al. (2020), Devhare et al. (2017)
	TAA	Gavage or intraperitoneal injection	3% TAA, 150–200 mg/kg	8–12 weeks	Hemodynamics, biochemical indices, and morphology are similar to humans	Easy to have toxic effects on genetic material transcription	Wu et al. (2013), Shahin et al. (2020)
	CCl_4_	Gavage	50% CCl4, 1 mL/kg	20 weeks	Low cost, high reliability, and good reproducibility	Low success rate, long cycle, and low mortality rate	Carson et al. (2018), Folch-Puy et al. (2016)
		Intraperitoneal injection	50% CCl4, 2 mL/kg	6 weeks		High success rate, short cycle, and high mortality rate	Koo et al. (2016)
Dietary model	Ethanol	Gavage	52% ethanol, 12 mL/kg	6–12 days	Suitable for alcoholic liver disease	Complex modeling method with low success rate	Liu et al. (2019), Liu et al. (2021)
	High-fat diet	Feeding	40% fat and 0.2% cholesterol, normal eating	12 weeks	Similar to human primary non-alcoholic fatty liver disease	Difficulty in controlling food intake, time, and significant individual differences in the model	Ni et al. (2017), Krenkel and Tacke (2017)
	Methionine-choline deficient diet	Feeding	60%–80% high-fat choline-deficient feed, normal eating	≥6 weeks	Can replicate severe human NASH clinical manifestations well, and fibrosis progresses rapidly	Only reflects human NASH indirectly, without insulin resistance, weight loss, and significant differences in results	Krenkel and Tacke (2017), Shi et al. (2011), Robertson and Rifkin (2016)
Autoimmune model	-	-	2% isoflurane, ligation of common bile duct	12–24 days	Simple operation and high modeling rate	Requires surgical operation	Khan and Marshall (2016)
	Pig serum	Intraperitoneal injection	Pig serum, 0.5 mL/animal	8–12 weeks	Similar to human primary liver fibrosis	Long modeling time and high animal mortality rate due to allergic reactions	Carthy (2018), Derynck and Budi (2019)
	Schistosoma mansoni cercariae	-	Schistosoma eggs, 18-20 eggs/animal	6 weeks	Schistosomiasis-induced liver fibrosis and cirrhosis	Limited application scope	Yoshida and Matsuzaki (2012)
Gene-modified model	Mdr2−/−	Gene knockout	-	3–6 months	Good reproducibility	Long cycle	Matsuzaki (2009), Feng et al. (2015)
	dnTGFβRⅡ	Genetic variation	-	8–12 weeks	Similar to human primary sclerosing cholangitis	No obvious disadvantages at present	George et al. (2020)
	Ae2a,b−/−	Gene knockout	-	1–9 months	Similar to human primary sclerosing cholangitis	Age limitation	Umbayev et al. (2014)

Chemical induction models (e.g., NMDA, TAA, CCl_4_, and ethanol) efficiently replicate acute/chronic liver injury: NMDA injection (0.5% NMDA/kg, 3–4 weeks) is stable and resembles human NASH but costly and highly toxic (Shi et al., 2011; George et al., 2020; Umbayev et al., 2014); TAA injection/oral (150–300 mg/kg, 8–12 weeks) morphologically mimics human conditions but has long duration and high mortality (Liu et al., 2020; Đurašević et al., 2021); CCl_4_ injection/oral (50% CCl_4_, 6 weeks) is reliable and low-cost, yet poses high toxicity and cirrhosis risk (Xiu et al., 2021; Lai et al., 2019); ethanol feeding (25% ethanol, 6–12 days) simulates alcoholic liver disease but is sensitive to steatosis (Jia et al., 2019; Bertola et al., 2013). Dietary models emphasize nutritional factors: high-fat diet (40% fat, 12 weeks) mimics human NAFLD but shows individual variability (Pokhrel et al., 2023; Tsuchida et al., 2018); MCD diet (high-fat, 2–6 weeks) induces severe injury but incompletely recapitulates human pathology (Li et al., 2021; Wang et al., 2022). Surgical and autoimmune models extend applications: pig serum injection (0.5 mL, 5–8 weeks) simulates primary biliary cholangitis but has limited responses (Li et al., 2018; Aikins et al., 2022; He et al., 2021); schistosomiasis infection (18–20 weeks) yields good fibrosis but is operationally complex (Toda et al., 2009). Genetic models (e.g., knockout variants, 8–12 weeks) are highly relevant and mimic human disease without major drawbacks (Hou et al., 2012; van Nieuwerk et al., 1997; Wang et al., 2018; Huang et al., 2021; Concepcion et al., 2015).

These models offer clinical relevance and reproducibility, but limitations include toxicity, prolonged timelines, and translational barriers. Future studies should integrate multi-model approaches to enhance bench-to-bedside bridging.

### 4.7 Targeted delivery and therapy

**FIGURE 11:**
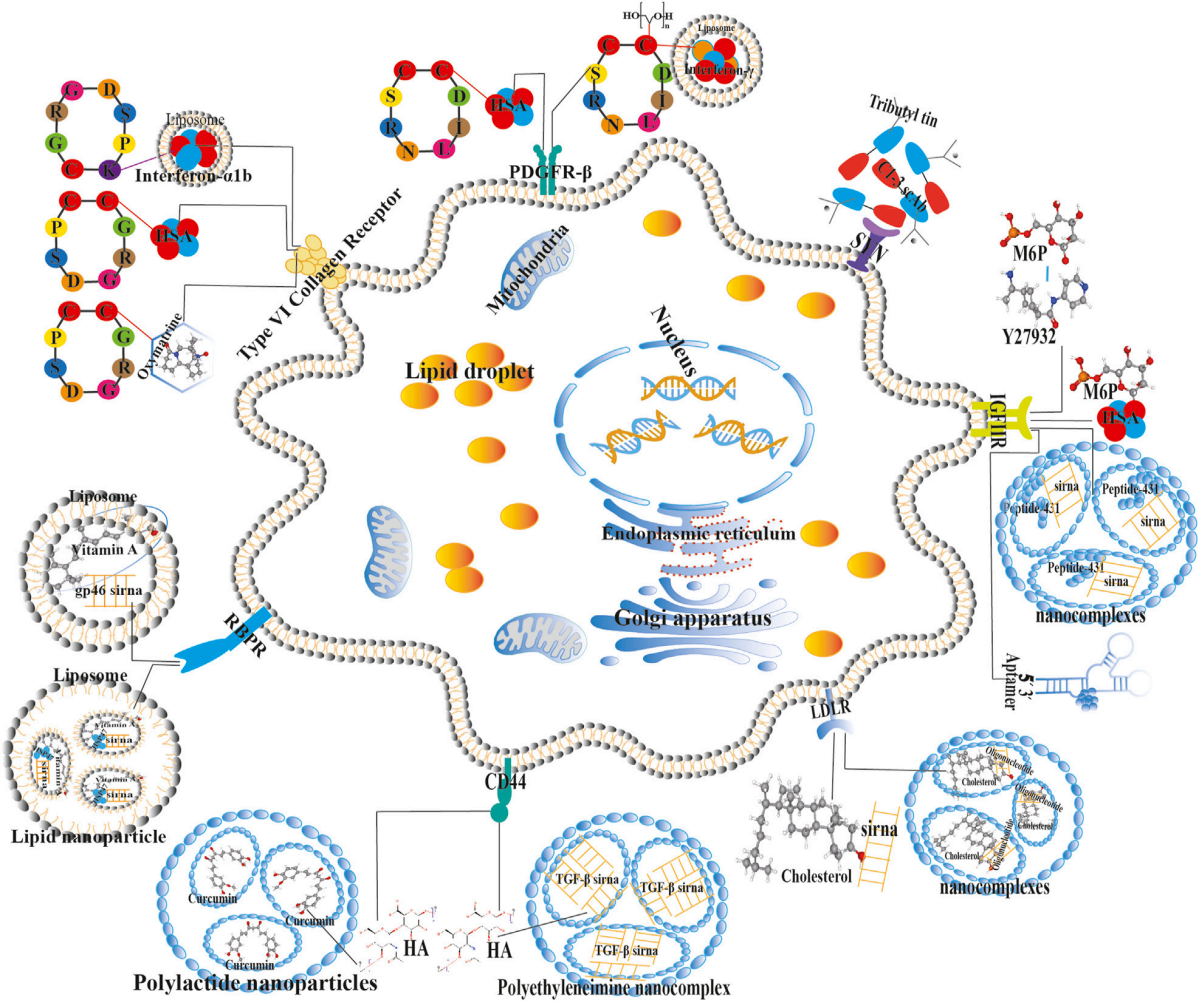
Schematic diagram of targeted drugs using different carriers for the treatment of LF initiated by HSCs.

Inhibition of HSCs is a crucial step in blocking LF progression. The effective delivery of traditional drugs in this process is influenced by the density of the ECM, the number of HSCs, and the specificity of membrane proteins. This is closely related to the type of targeted drug, delivery vector, cell receptor recognition, endocytosis, and intracellular drug release processes (Li et al., 2015). Human serum albumin (HSA) and HSCs play a positive role in improving LF treatment. Our further analysis of HSA recognition, delivery, and treatment by HSCs has revealed that after the occurrence of LF, Type VI collagen receptor, PDGF receptor-β (PDGFR-β), and Insulin-like growth factor II receptor (IGF-IIR) are abnormally activated (Figure 11). Research has shown that the cyclic arginylglycylaspartic acid (RGD) peptide CGRGDSPC (* denotes the cyclizing cysteine residue) ligand, often coupled with HSA, can be specifically recognized and internalized into the cytoplasm by Type VI collagen receptors on the surface of HSCs, thereby reducing the production of Type VI collagen (Beljaars et al., 2000). The cyclic peptide CSRNLIDC can specifically bind to HSA and be recognized by PDGFR-β, inhibiting the proliferation of HSCs (Beljaars et al., 2003). Mannose 6 phosphate (M6P), a natural ligand for IGFIIR, binds to HSA and is specifically recognized by HSCs, accelerating targeted drug endocytosis (Ye et al., 2006).

Small interfering RNA (siRNAs) are newly discovered therapeutic agents that regulate the expression of specific genes. Following HSCs activation, LF occurs, and the surface retinol-binding protein receptor (RBPR), low-density lipoprotein receptor (LDLR), IGFIIR, and Cluster of differentiation 44 (CD44) are activated (Zhang et al., 2015). Research has shown that gp46 siRNA can bind to retinol and be encapsulated in liposomes, which then form a complex with retinol-binding protein (RBP) and are converted into retinyl esters by retinol acyltransferase (LRAT) (Blaner et al., 2009; Senoo et al., 2013). Recognition by RBPR leads to internalization of the complex, effectively inhibiting the expression of gp46 (Sato et al., 2008). Using the same method, heat shock protein 47 (HSP47) was delivered into HSCs using nanoliposomes to regulate the expression of this gene. The accumulation of cholesterol makes HSCs sensitive to TGF-β, exacerbating the occurrence of LF (Teratani et al., 2012). This was closely related to the targeted binding of LDLR. When cholesterol is coupled with a streptavidin-based siRNA nanocomplex and expressed internally in HSCs (Shukla et al., 2013), it inhibits the occurrence of LF. The effective combination of Peptide-431 and anti-fibrotic siRNA formed a specific nanocomplex ligand for IGFIIR (Chen et al., 2015). Simultaneously, a combination of aptamers (Chen et al., 2017) and siRNA can also inhibit HSCs activation. Hyaluronic acid (HA) is the main ligand of CD44 and is an essential component of the ECM. HA coupled with TGF-β siRNA encapsulated in polyethylenimine nano-complexes specifically binds to CD44 and enters HSCs via endocytosis, significantly reducing LF.

Interferon (INF) is often associated with immune responses and antiviral treatments. After LF is induced by the Hepatitis B/C Virus, the Type VI collagen receptor and PDGFR-β on the surface of HSCs are activated. The study found that the cyclic RGD peptide CGRGDSPK targeted ligand coupled with INF-α1b liposomes can be recognized explicitly by Type VI collagen receptor on the surface of HSCs (Du et al., 2007), and rapidly accumulated inside the cells to exert anti-fibrotic effects caused by viral hepatitis. The cyclic peptide CSRNLIDC, which is coupled with liposome-encapsulated INF-γ using polyethylene glycol as a linker (Li et al., 2012), is recognized by PDGFR-β on the surface of HSCs, internalized into the cells, and exhibits strong antiproliferative effects (Li Q. et al., 2017).

The active components of traditional Chinese medicine, oxymatrine, and curcumin, have potent anti-inflammatory effects. Research has found that oxymatrine, after polymerization with cyclic RGD peptides, is recognized by the Type VI collagen receptor (Yang et al., 2014), significantly inhibiting the activation and proliferation of HSCs and reducing the expression of intracellular α-SMA and collagen. Curcumin encapsulated in HA-modified polylactic acid nanoparticles is recognized by CD44 and induces apoptosis of activated HSCs (Chen et al., 2016). Synaptophysin (SYN), a component of endocytic vesicles, can bind the ligand combining single-chain antibody (scAb) C1-3 with tributyl tin to the SYN receptor (Douglass et al., 2008), specifically via endocytosis, for the treatment of nonalcoholic steatohepatic LF. The Rho-kinase inhibitor (Xu et al., 2023)-(R)-trans-4-(1-aminoethyl)-N-(4-pyridyl) cyclohexanecarboxamide dihydrochloride (Y27632), coupled with the specific ligand M6P (van Beuge et al., 2011) can be recognized by IGFIIR on the surface of HSCs, thereby reducing collagen deposition in the ECM.

### 4.8 Integrating mechanisms, patents, and clinical translation in HSC-targeted anti-fibrotic therapy

Analyses of keyword co-occurrence and clustering delineate the central research trajectory of HSCs biology within LF. High-frequency and high-centrality terms converge on LF, HSCs, TGF-β signaling, gene and RNA expression, HSCs activation, proliferation, and apoptosis, indicating a sustained focus on the causal chain linking mechanisms, expression changes, and cellular phenotypes. Temporally, investigations of HSCs activation mechanisms preceded studies of inhibitory pathways, after which targeted delivery and therapeutic strategies emerged as the leading edge. Keyword bursts emphasize long-standing reliance on rodent models, frequently employing carbon tetrachloride injury, and note parallel signals related to HCC. Together, these patterns support an evolutionary logic that moves from defining activation, to developing inhibitory strategies, and finally to intervention and delivery *in vivo*.

TheIPC structure is concordant with this knowledge landscape. Therapeutic-use classifications are dominant, with A61P 1/16 (drugs for liver or gallbladder disorders) capturing most inventions and underscoring direct clinical intent. A61K 31/(medicinal preparations containing organic active ingredients) remains prominent, indicating the continued centrality of small-molecule candidates and aligning with earlier mechanism-focused drug discovery. The presence of A61K 9/00 (specialized dosage forms) and A61K 45/06 (combination or “cocktail” regimens) reflects a patenting pathway that leverages pharmaceutical sciences and rational combinations to improve hepatic delivery, control exposure, and increase effect sizes. Nucleic acid–based regulation (C12N 15/113) and antibody inventions (C07K 16/18) signal an accumulation of intellectual property around molecularly precise interventions, whereas patents involving mammalian materials and undifferentiated cells (A61K 35/28 and C12N 5/071) echo regenerative and reparative approaches. Overall, the patent corpus is expanding from single-entity composition claims toward multi-modal platforms spanning formulation engineering, nucleic acids, antibodies, and delivery, with emerging alignment to diagnostic and method-of-use dimensions.

Temporal trends and clinical trial structures have, to some extent, validated but also constrained this innovative pathway. Early trials primarily evaluated small-molecule monotherapies, often targeting the renin-angiotensin system to inhibit angiotensin-driven HSCs activation and collagen synthesis—this strategy aligned with contemporaneous etiological contexts such as hepatitis C and resonated with the proliferation of A61K31/patents, where high-frequency filings directly propelled the clinical dominance of small-molecule candidates. Mid-term studies shifted toward cell therapies and biologics, which both suppress HSCs activation and promote liver regeneration; however, manufacturing consistency, immunological safety, and efficacy reproducibility emerged as key barriers. For instance, the lower proportions of A61K35/28 (involving mammalian materials such as stem cells or hepatocytes; frequency 5%, 4.1%) and C12N5/071 (undifferentiated cells such as hepatocytes; frequency 5%, 4.1%) reflect insufficient intellectual property accumulation for regenerative approaches, thereby limiting the scalability and reproducibility of mid-term trials. Recent trials demonstrate greater diversity and combinatorial potential (including nutritional/metabolic interventions and bile acid-related drugs) while predominantly employing randomized controlled designs, aligning with the emergence of multimodal strategies in keyword clustering. For example, in MASH-related fibrosis trials, combination therapies (e.g., bile acid derivatives with HSCs inhibitors) significantly improved fibrosis scores; these advancements directly derive from keyword co-occurrences in early mechanistic studies (e.g., TGF-β and ECM remodeling) and are supported by A61K45/06 patents (combination regimens; frequency 11%, 9.1%), whose growth validates the clinical transition to “cocktail” therapies. Clinical trial volumes peaked around 2020 (approximately 10–15 per year), with Phase 2 studies comprising the highest proportion, underscoring how trends drive progression from monotherapy to multimodal approaches while exposing Phase 3 translational bottlenecks. Nevertheless, median sample sizes remain modest, follow-up durations are often limited, surrogate endpoints exhibit variable sensitivity, and termination rates are substantial, indicating that signal detectability and statistical power continue to constrain translational efficiency. Concurrently, fluctuations and recent slowdowns in patent applications may reflect a shift from expansion to refinement, prioritizing specificity, implementability, and clinical falsifiability. Overall, these trends map the incremental advancement of anti-fibrotic agents from fundamental mechanisms to clinical validation but also highlight translational bottlenecks, such as elevated termination rates for HSC-targeted nanotherapies in post-2024 trials.

Synthesizing evidence across mechanisms, patenting, and trials suggests several defining features and forward directions for anti-fibrotic therapeutics. First, mechanisms will continue to anchor development, with targeted interventions directed at TGF-β signaling, ECM remodeling, receptor-mediated transduction, and apoptosis, while placing greater emphasis on dual strategies that inhibit HSCs activation and promote reversion to quiescence (deactivation). Second, therapeutic modalities will remain multi-modal. Small molecules retain a foundational role due to oral availability and accessibility; nucleic acid therapeutics and monoclonal antibodies offer selective pathway modulation; and cellular therapies carry potential for regeneration and repair. Third, pharmaceutics and delivery science will be pivotal for enhancing hepatic uptake and minimizing systemic exposure. Ligand-targeted delivery, nanomedicine formulations, and specialized dosage forms should be co-designed with clinical stratification schemas and clearly defined use scenarios to optimize benefit–risk profiles.

Clinical development should strengthen the mechanism–pharmacodynamic–endpoint continuum. Early-phase studies would benefit from incorporating non-invasive imaging biomarkers such as magnetic resonance elastography and corrected T1 mapping, as well as serum collagen neo-epitope markers such as PRO-C3, and, where appropriate, portal hemodynamic measures such as the hepatic venous pressure gradient (HVPG). Trials should prioritize populations with homogeneous etiology and well-defined disease stage, consider metabolic and inflammatory control as background therapy, and layer direct anti-fibrotic interventions using rational combinations. Adaptive or factorial designs can improve sample efficiency and decision quality while accommodating heterogeneity.

### 4.9 Literature gaps and future directions in liver fibrosis research

This bibliometric analysis highlights the progression in liver fibrosis research from mechanistic insights to targeted therapies, while revealing several critical gaps. These include suboptimal bridging between mechanism-oriented clusters (e.g., TGF-β signaling and ECM crosslinking) and clinical application clusters in co-occurrence networks, as well as low efficiency in patent-to-clinical translation. Although the literature comprehensively delineates core fibrogenesis mechanisms (e.g., hepatic stellate cell activation), large-scale cohort studies integrating multi-source data are notably absent, resulting in inadequate clinical validation of emerging agents, low translational rates, and fragmented research that impedes the transition from preclinical stages to randomized controlled trials.

Another key gap pertains to deficiencies in adverse event management and personalized therapeutic strategies, coupled with a lack of standardization for noninvasive assessment tools. While targeted delivery patents have proliferated, systemic toxicity evaluations remain fragmented, with a paucity of comparative studies assessing the impact of genotypic variations on therapeutic efficacy, thereby restricting the application of high-potential therapies in multi-organ fibrosis. Clinical guidelines emphasize tools such as FIB-4 and vibration-controlled transient elastography (VCTE), yet associated research inadequately incorporates emerging imaging modalities (e.g., hyperspectral imaging), particularly evidencing voids in pediatric metabolic dysfunction-associated steatotic liver disease (MASLD). These gaps exacerbate shortcomings in multidisciplinary collaboration and real-world evidence generation, constraining the global applicability of guideline stratification.

Currently, hepatic stellate cell (HSC)-targeted antifibrotic therapies are transitioning from single-molecule approaches to integrated, context-adaptive platform systems. Guided by the integrated analysis of knowledge maps, patents, and clinical data, future high-value research and development will focus on mechanism-driven precision interventions targeting core domains such as TGF-β signaling, extracellular matrix (ECM) remodeling, receptor transduction, and apoptosis, while emphasizing dual strategies to inhibit HSC activation and promote deactivation. These pathways should incorporate optimized delivery technologies to enhance hepatic uptake and minimize systemic exposure; integrate patient stratification strategies addressing homogeneous etiologies and well-defined disease stages; and rely on robust evidence chains, including strengthening mechanism-efficacy-endpoint linkages, prioritizing multicenter randomized controlled trials with adaptive designs, and incorporating real-world evidence for long-term efficacy assessment.

Cluster analysis indicates active immune mechanisms but suboptimal integration with guideline stratification, thus recommending multicenter randomized controlled trials that integrate artificial intelligence-driven biomarkers to evaluate the long-term efficacy of novel inhibitors in MASLD patient cohorts; concurrently, interdisciplinary platforms should be established to generate real-world evidence supporting the generalization of patent innovations across diverse populations. Future efforts should prioritize the development of advanced targeted systems (e.g., CRISPR-edited nanoplatforms for quantifying side effect reduction through phase II trials and exploring combinations with immunotherapies) as well as noninvasive innovations (e.g., AI-enhanced hyperspectral imaging for threshold standardization). These initiatives are anticipated to pioneer novel therapeutic paradigms, improve overall survival rates, and enhance the translational efficiency and clinical accessibility of antifibrotic therapies.

This bibliometric analysis was conducted exclusively within English-language literature databases (e.g., Web of Science, PubMed, Scopus), which may introduce language and regional biases by omitting relevant studies published in non-English journals—particularly those concerning traditional Chinese medicine, regional epidemiological patterns, or local therapeutic practice. Consequently, the global distribution of research effort, citation impact, and thematic emphasis may be under-represented, and culturally specific knowledge systems could be inadvertently marginalized. Future analyses should therefore incorporate Chinese, Japanese, Korean, and other regional databases (e.g., CNKI, Wanfang, J-STAGE, KISS) to capture a more inclusive, culturally balanced landscape of anti-fibrotic research.

## 5 Conclusion

By integrating bibliometrics, IPC patents, and clinical-trial trends, we map an HSC - centered antifibrotic trajectory. Research converges on TGF-β signaling, ECM remodeling, and HSC activation, with targeted delivery as the leading frontier. Future efforts should prioritize mechanism-driven precision interventions, coupled with patient stratification and validated noninvasive endpoints, to accelerate translation from research to regulatory approval and patient access.

## Data Availability

Publicly available datasets were analyzed in this study. This data can be found here: The datasets analyzed in this study are publicly available from the following sources without specific accession numbers, as they consist of bibliographic and patent records retrieved via the search queries detailed in the Methods section. Data can be reproduced using the provided search strategies. - Web of Science Core Collection (WoSCC): https://www.webofscience.com (Clarivate Analytics repository; access may require institutional subscription). - Scopus: https://www.scopus.com (Elsevier repository; access may require institutional subscription). - PubMed: https://pubmed.ncbi.nlm.nih.gov (National Library of Medicine repository; free public access). - ClinicalTrials.gov: https://clinicaltrials.gov (U.S. National Library of Medicine repository; free public access). - Innojoy Patent Search Engine: https://www.innojoy.com (or equivalent patent database; free basic access, premium features may require subscription). For further details, contact the corresponding author.
