# Supplementary material for: From mechanisms to anti-fibrotic drugs in hepatic stellate cell research: a global bibliometric analysis with patent and clinical perspectives (2000–2025)
Source: Front Pharmacol. 2025 Dec 10;16:1734449. doi: 10.3389/fphar.2025.1734449 (PMC12727573; doi:10.3389/fphar.2025.1734449)
Supplement: Supplementary file 1 [file Supplementaryfile1.docx]

Supplementary Material

# Supplementary Tables

Table S1 Top ten institutions in the research field of initiation or improvement of LF by HSCs.

| **Rank** | **Institutions** | **Countries** | **Years** | **Centrality** | **Publications** |
| --- | --- | --- | --- | --- | --- |
| 1 | Anhui Medical University | China | 2009 | 0.02 | 45 |
| 2 | Shanghai Jiao Tong University | China | 2000 | 0.07 | 41 |
| 3 | Nanjing Medical University | China | 2013 | 0.11 | 34 |
| 4 | Fudan University | China | 2000 | 0.04 | 32 |
| 5 | Capital Medical University | China | 2009 | 0.08 | 26 |
| 6 | Nanjing University of Chinese Medicine | China | 2013 | 0.03 | 25 |
| 7 | Chinese Academy of Sciences | China | 2005 | 0.10 | 24 |
| 8 | Sun Yat Sen University | China | 2011 | 0.03 | 24 |
| 9 | University of California System | USA | 2000 | 0.09 | 22 |
| 10 | Icahn School of Medicine at Mount Sinai | USA | 2002 | 0.18 | 19 |

Table S2 Top ten authors in the field of LF initiated or improved by HSCs.

| **Rank** | **Authors** | **Publications** | **Years** | **Institutes** | **Countries** | **Citations** | **Average citation** |
| --- | --- | --- | --- | --- | --- | --- | --- |
| 1 | Zhang, Feng | 19 | 2016 | Nanjing University of Chinese Medicine | China | 821 | 43.21 |
| 2 | Li, Jun | 18 | 2014 | Anhui Medical University | China | 635 | 35.28 |
| 3 | Zheng, Shizhong | 16 | 2013 | Nanjing University of Chinese Medicine | China | 562 | 35.13 |
| 4 | Huang, Cheng | 13 | 2016 | Anhui Medical University | China | 348 | 26.77 |
| 5 | Shao, Jiangjuan | 12 | 2016 | Nanjing University of Chinese Medicine | China | 476 | 39.67 |
| 6 | Zhang, Zili | 10 | 2013 | Nanjing University of Chinese Medicine | China | 430 | 43.00 |
| 7 | Brenner, David A | 7 | 2006 | University of California System | USA | 1381 | 197.29 |
| 8 | Li, Rui | 6 | 2020 | Sichuan University | China | 219 | 36.50 |
| 9 | Friedman, Scott L. | 5 | 2007 | Icahn School of Medicine at Mount Sinai | USA | 1894 | 378.80 |
| 10 | Meng, Xiaoming | 5 | 2015 | Anhui Medical University | China | 167 | 33.40 |

Table S3 Top ten co-cited authors in the field of LF initiated or improved by HSCs.

| **Rank** | **Co-cited authors** | **Publications** | **Years** | **Institutes** | **Countries** | **Centrality** |
| --- | --- | --- | --- | --- | --- | --- |
| 1 | FRIEDMAN SL | 543 | 2000 | Icahn School of Medicine at Mount Sinai | USA | 0.05 |
| 2 | BATALLER R | 349 | 2001 | University of Barcelona | Spain | 0.10 |
| 3 | KISSELEVA T | 253 | 2010 | University of California System | USA | 0.04 |
| 4 | TSUCHIDA T | 240 | 2018 | Icahn School of Medicine at Mount Sinai | USA | 0.02 |
| 5 | HIGASHI T | 170 | 2018 | Hokkaido University | Japan | 0.01 |
| 6 | MEDERACKE I | 168 | 2015 | Hannover Medical School | Germany | 0.01 |
| 7 | IREDALE JP | 143 | 2001 | University of Edinburgh | UK | 0.08 |
| 8 | SEKI E | 139 | 2012 | Cedars-Sinai Medical Center | USA | 0.04 |
| 9 | HERNANDEZ-GEA V | 132 | 2012 | University of Barcelona | Spain | 0.05 |
| 10 | SCHUPPAN D | 131 | 2001 | Johannes Gutenberg University of Mainz | Germany | 0.04 |

Table S4 Keywords with a frequency of ≥20 in the field of LF caused or improved by HSCs.

| **Rank** | **Keywords** | **Frequency** | **Centrality** | **Rank** | **Keywords** | **Frequency** | **Centrality** |
| --- | --- | --- | --- | --- | --- | --- | --- |
| 1 | liver fibrosis | 702 | 0.04 | 22 | cancer | 47 | 0.04 |
| 2 | hepatic stellate cells | 515 | 0.06 | 23 | in vitro | 46 | 0.06 |
| 3 | expression | 351 | 0.12 | 24 | pathogenesis | 45 | 0.05 |
| 4 | mechanisms | 246 | 0.07 | 25 | rats | 45 | 0.05 |
| 5 | activation | 214 | 0.13 | 26 | nf kappa b | 42 | 0.05 |
| 6 | tgf beta | 189 | 0.10 | 27 | growth factor | 42 | 0.07 |
| 7 | proliferation | 135 | 0.09 | 28 | autophagy | 40 | 0.04 |
| 8 | apoptosis | 135 | 0.11 | 29 | rat liver | 39 | 0.05 |
| 9 | injury | 110 | 0.09 | 30 | carbon tetrachloride | 36 | 0.04 |
| 10 | inhibition | 99 | 0.13 | 31 | differentiation | 32 | 0.04 |
| 11 | pathway | 88 | 0.06 | 32 | fat storing cells | 32 | 0.05 |
| 12 | inflammation | 83 | 0.05 | 33 | animal model | 31 | 0.03 |
| 13 | cirrhosis | 77 | 0.13 | 34 | myofibroblasts | 29 | 0.04 |
| 14 | liver disease | 75 | 0.06 | 35 | nonalcoholic steatohepatitis | 26 | 0.05 |
| 15 | mice | 70 | 0.03 | 36 | target | 24 | 0.02 |
| 16 | receptor | 65 | 0.09 | 37 | gene | 22 | 0.01 |
| 17 | oxidative stress | 64 | 0.07 | 38 | alpha-smooth muscle actin | 22 | 0.04 |
| 18 | extracellular matrix | 57 | 0.11 | 39 | therapy | 22 | 0.01 |
| 19 | hepatocellular carcinoma | 55 | 0.08 | 40 | angiogenesis | 22 | 0.04 |
| 20 | progression | 47 | 0.06 | 41 | collagen | 21 | 0.06 |
| 21 | in vivo | 47 | 0.10 | 42 | metabolism | 20 | 0.01 |
